# Ocean temperature drove changes in the mesopelagic fish community at the edge of the Pacific Warm Pool over the past 460,000 years

**DOI:** 10.1126/sciadv.adf0656

**Published:** 2023-07-07

**Authors:** Chien-Hsiang Lin, Chih-Lin Wei, Sze Ling Ho, Li Lo

**Affiliations:** ^1^ Biodiversity Research Center, Academia Sinica, Greenhouse Building R246, 128 Academia Road, Sec. 2, Nankang, Taipei 11529, Taiwan.; ^2^ Institute of Oceanography, National Taiwan University, Taipei 10617, Taiwan.; ^3^ Department of Geosciences, National Taiwan University, Taipei 10617, Taiwan.; ^4^ Research Center for Future Earth, National Taiwan University, Taipei 10617, Taiwan.

## Abstract

Field observations and theoretical modeling suggest that ongoing anthropogenic ocean warming will lead to marine ecosystem degradation. Mesopelagic fish are a fundamental component of the pelagic ecosystem, and their role in linking the surface- and deep-ocean ecosystems is essential for the biological carbon pump. However, their response to a warmer ocean is unconstrained because of data scarcity. Using extraordinarily well-preserved fish otoliths, we reconstruct a continuous mesopelagic fish community record in the Pacific Warm Pool region over 460,000 years. Fish production and diversity followed hump-shaped temperature gradients, with lower tipping point temperatures for the diversity than the production by ~1.5° to 2.0°C. During warmer-than-present interglacial periods, both production and diversity declined drastically. Our findings imply that the temperature-sensitive mesopelagic fish community at the southwestern margin of the Pacific Warm Pool, and possibly other hydrographically similar regions, may be especially affected if ocean warming continues unabated in the future.

## Teaser

Deep-sea fossils reveal cyclic fluctuations in fish community structure over four glacial cycles.

## INTRODUCTION

Mesopelagic fishes efficiently bridge the trophic levels in marine ecosystems via their diel vertical migration, serving as a “biological pump” in the carbon cycle (*1*–*4*). They constitute the bulk of midtrophic biomass; therefore, fluctuations in their abundance have profound impacts on the associated ecosystems (*5*, *6*) and fishery landings (*7*). In modern day, mesopelagic fish biomass appears to couple with primary productivity and ocean temperature (*8*). Thus, fossils from past warm periods may provide constraints to the response of the fish community to a warmer-than-present ocean.

Fish remains such as otoliths provide insightful windows into the spatiotemporal dynamics of regional fish communities in the past (*9*–*11*). However, studies based on deep-sea sediment core materials rarely investigate fish otoliths (*12*), despite their long history in paleontological research (*9*, *10*), ubiquity in marine sediments (*13*, *14*), and high taxonomic resolution (*15*). Recent studies have used fish fossils to investigate the response of fish communities to large climatic shifts during the Eocene/Oligocene boundary (*16*) and Marine Isotope Stage 5e (MIS 5e) in the Humboldt Current System (*17*). However, there is a dearth of continuous fish fossil records on glacial-interglacial (G-IG) time scales, especially in the Pacific Warm Pool, often regarded as a biodiversity hotspot (*18*).

Here, we reconstruct the history of fish community structure over the past 460 thousand years (ka) by generating continuous otolith time series from Ocean Drilling Program (ODP) Hole 1115B, located in the Solomon Sea at the southwestern margin of the Pacific Warm Pool (*19*). The shallow water depth (1149 m) at this pelagic site not only preserves excellent carbonate microfossils (including aragonites) but also provides well-constrained age model by means of calcite microfossil-based oxygen isotope stratigraphy and biostratigraphic events (*19*). The Pacific Warm Pool contains the largest body of warm water in the global ocean (*20*); thus, data from past warm periods here may provide insights into how the fish community may respond to projected future warming. We calculated the abundance and diversity of fish otoliths and compared them to hydroclimatic variables. Our data show that temperature exerts a strong control on mesopelagic fish community structure at the southwestern margin of the Pacific Warm Pool on G-IG time scales.

## RESULTS

### Otolith-derived fish production and diversity

**Fig. 1.**
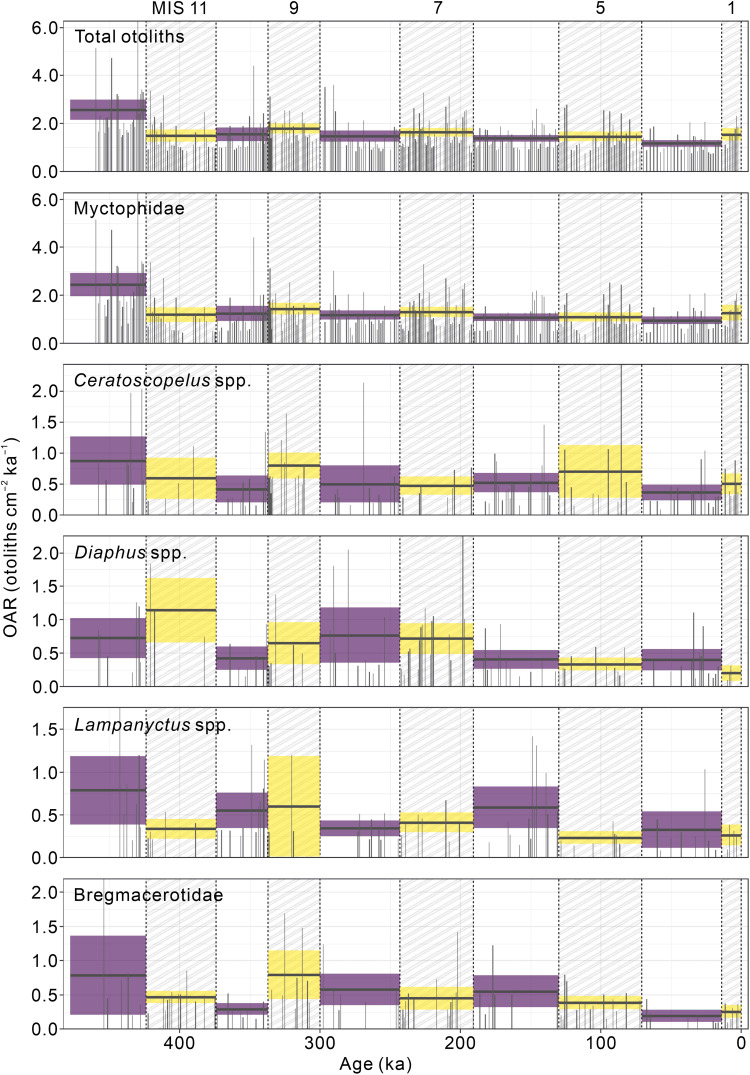
OAR of major taxa (number of otoliths > 80) over the past 460 ka. Hatched rectangles indicate interglacial periods (MISs 1, 5, 7, 9, and 11 on the upper *x* axis). The black horizontal lines show the mean, and the shaded area show the 95% confidence intervals.

A total of 1130 otoliths were extracted from 343 sediment samples. Except for juvenile or worn specimens (*n* = 131, 11.6%), they were identified to 28 taxa belonging to 12 families (data S1). The otolith record at site ODP 1115B is dominated by mesopelagic fishes, among which by the family Myctophidae (73.7%) and specifically by its genera *Ceratoscopelus*, *Diaphus*, and *Lampanyctus* (Fig. 1 and data S1). Another abundant family is the epi- to mesopelagic Bregmacerotidae (9.1%). Otoliths of *Cyclothone*, the most abundant vertebrate in the ocean, were absent in the record. Similar observation was previously reported by Jones and Checkley Jr. (*12*) for the Santa Barbara Basin. We speculate that the otoliths of *Cyclothone* and possibly all other small-sized otoliths might be more prone to dissolution or lost during sample processing because of their small size (Materials and Methods).

We use otolith accumulation rate (OAR, number of otoliths cm^−2^ ka^−1^) to account for changes in sedimentation rate and sediment density (fig. S4) along the sediment core to estimate the fish production, and Hill numbers (*21*) for fish diversity (Materials and Methods and Supplementary Text). Hill numbers are widely applied in ecology to quantify diversity and reported in three orders (*q* = 0 to 2) (*22*). Unlike commonly applied unitless diversity indices, such as Shannon and Simpson indices, Hill numbers integrate species richness and abundance into hypothetical numbers of equally abundant species. Therefore, Hill numbers provide a unified framework to quantify diversity as numbers equivalents (*23*). For example, −3 in Hill number of order *q* = 1 (^1^*D*) means that the number of abundant species decline by 3. The abundant species are usually well adapted to the environmental condition. A decline in the number of abundant species could indicate an impact on a specific community.

**Fig. 2.**
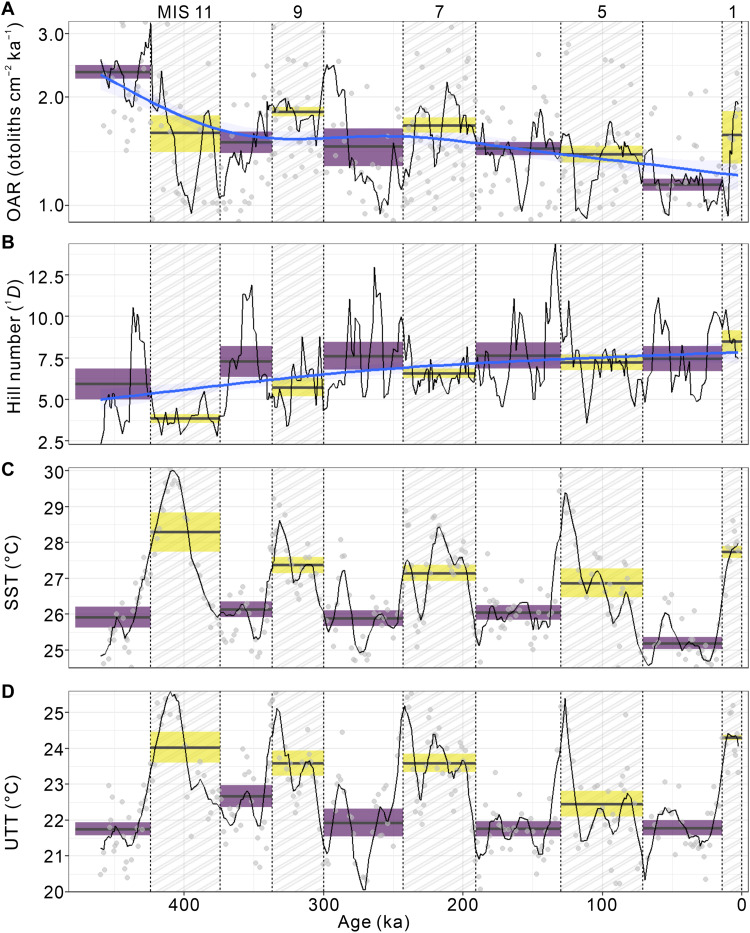
Fish production, diversity, and seawater temperature variabilities at the southwestern margin of the Pacific Warm Pool over the past 460 ka. (**A**) OAR, (**B**) Hill number of order *q* = 1 (^1^*D*, Shannon diversity), (**C**) sea surface temperature (SST), and (**D**) upper thermocline temperature (UTT; ~110-m depth). Raw data are represented by gray dots, and data smoothing (black lines) is calculated using a 10-ka moving window (Materials and Methods). Hatched rectangles indicate interglacial periods (MISs 1, 5, 7, 9, and 11 on the upper *x* axis). The black horizontal lines indicate the mean, and the shaded areas show the 95% confidence intervals. (A) and (B) from ODP Hole 1115B, (C) and (D) from MD05-2925. SST data are from Lo *et al.* (*25*), while UTT data are from Lo *et al.* (*26*). The blue line (*P* < 0.05) and shaded areas in (A) and (B) show generalized additive model (GAM) fits on smoothed OAR and ^1^*D* using a moving-window approach with 95% confidence intervals.

We then applied a 10-ka moving window smoothing (Materials and Methods) on OAR and Hill numbers to visually enhance the G-IG changes in the time series (Fig. 2). Along the record, the mean OARs during the interglacial periods range from 1.1 to 1.8 (otoliths cm^−2^ ka^−1^), with gradual decrease from MISs 9 to 5 but stays high during MIS 1 (Fig. 2A). The mean OARs are stable at 1.4 to 1.5 (otoliths cm^−2^ ka^−1^) during the glacial periods (MISs 10, 8, and 6), except that the lowest value (~1.1 otoliths cm^−2^ ka^−1^) observed during the last glacial period (Fig. 2A). High OARs (2.3 otoliths cm^−2^ ka^−1^) and low Hill numbers occur during MIS 12, but these are likely an artifact as our truncated records do not include the entire stage. During the interglacial periods, the mean Hill numbers (*q* = 1) gradually increase from 3.8 to 8.5 (species) from MISs 11 to 1, whereas the mean Hill numbers are stable (7.5 to 7.6 species) during the glacial periods (Fig. 2B).

**Fig. 3.**
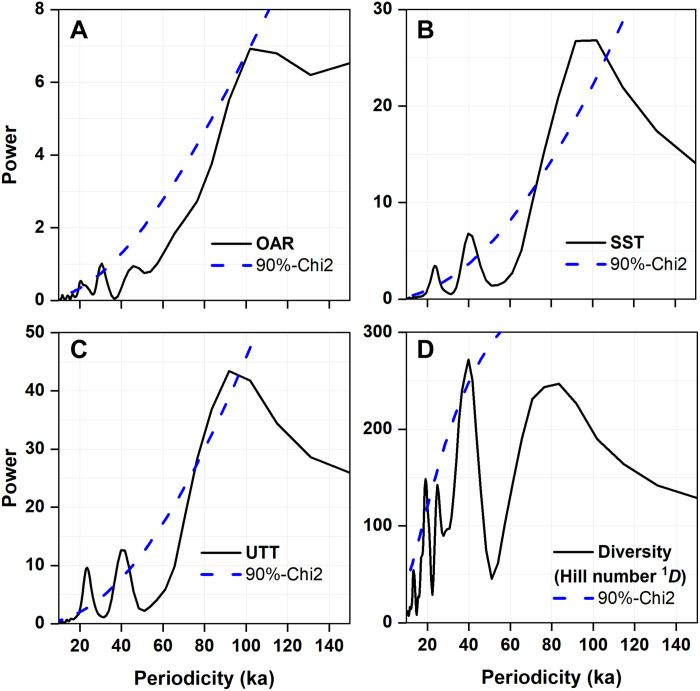
Power spectra of fish production, diversity, and seawater temperatures. (**A**) OAR, (**B**) SST, (**C**) UTT, and (**D**) fish diversity [Hill number = (^1^*D*)]. All data were smoothed using a 10-ka moving window (Materials and Methods). (A) and (D) Data from ODP Hole 1115B. (B) and (C) Data from core MD05-2925. The blue dashed line is 90% confidence line (*60*).

**Fig. 4.**
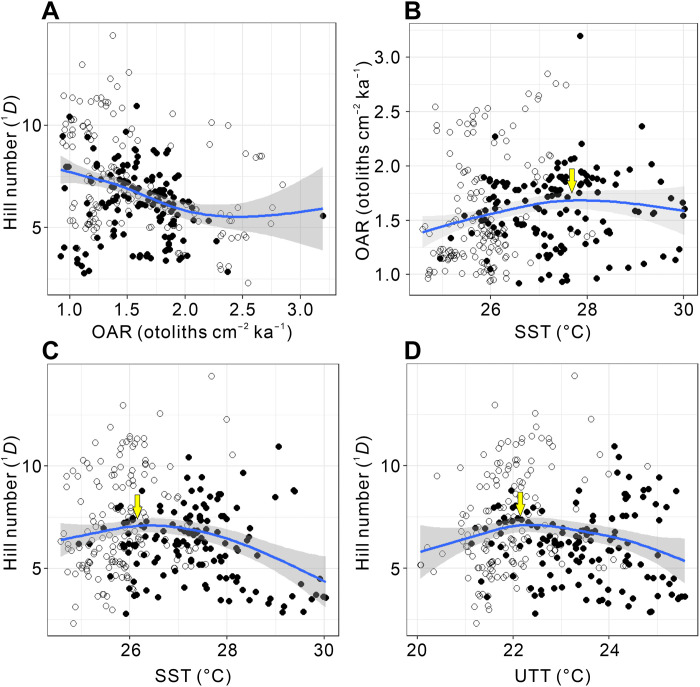
Relationships among fish production, diversity, and seawater temperatures. (**A**) Hill numbers of order *q* = 1 (^1^*D*, Shannon diversity) against OAR, and (**B**) OAR against SST, and Hill numbers (^1^*D*) against (**C**) SST and (**D**) UTT. Data were fitted by GAM. Only significant trend lines (*P* < 0.05) are shown, and the gray shaded areas show 95% confidence intervals. The open symbols indicate glacial periods, while closed symbols show interglacial periods. Arrows (yellow) in (B) to (D) indicate peaks of hump-shaped gradients.

The smoothed records show that the mesopelagic fish are more abundant but less diverse during the interglacial periods and vice versa (Fig. 2, A and B, and fig. S5). A clear 100-ka cyclicity is also detected for OAR (Fig. 3A), although it is less evident for diversity (Fig. 3D). Generalized additive model (GAM) indicates that diversity significantly declines (*P* < 0.05) with increasing OAR (Fig. 4A and fig. S6).

### Seawater temperature and fish community

We compare our mesopelagic fish community changes during the past 460 ka to productivity and seawater temperature records to examine whether these factors also apply on a longer time scale in the region. Because of the high biogenic carbonate concentration (>60%) of the core (*19*), we used the accumulation rate of the coarse sands (SAR; Materials and Methods and fig. S7A) as a proxy for net export productivity (*24*). In addition, total organic carbon (TOC) data across two selected glacial cycles were also generated as an additional proxy for productivity (fig. S8). We find no relationship between the OAR and productivity proxies (SAR and TOC) (Fig. 2A and figs. S7 and S8).

Temperature proxy records at the southwestern margin of the Pacific Warm Pool are available from a nearby site MD05-2925 (~10 km apart, fig. S9D and data S2) (*25*, *26*). Oceanographic setting at both sites (ODP 1115B and MD05-2925) are comparable (fig. S9), and the δ^18^O-based age models from both sites are visually aligned to the global benthic foraminiferal oxygen isotope stack (fig. S9) [LR04, (*27*)]. Both sea surface temperature (SST) and upper thermocline temperature (UTT) data from core MD05-2925 show almost identical temporal patterns (Figs. 2, C and D, and 3, B and C), with higher (lower) temperatures during interglacial (glacial) periods. They also correlate well with the LR04 reference stack (fig. S10) and, to a lesser extent, to the OAR record (Fig. 2A).

Fitted with GAM, Hill numbers and OAR show initial increase then decrease after maximum (hump-shaped gradients) with SST, where they peak at ~26° and ~28°C, respectively (Fig. 4, B and C, and fig. S11). The Hill numbers show a hump-shaped pattern with UTT that peaks at ~22°C (Fig. 4D and fig. S11), but OAR shows a double hump-shaped pattern with UTT that peaks at 21° and 23.5°C (fig. S11B). The main reason for the difference in the aforementioned hump-shaped patterns between OAR and UTT and between OAR and SST may arise from a smaller range of G-IG temperature differences in UTT. The model also has a larger scatter between OAR and UTT in the lower end of the thermal range (fig. S11B).

### Resilient taxa and possible future scenarios

**Fig. 5.**
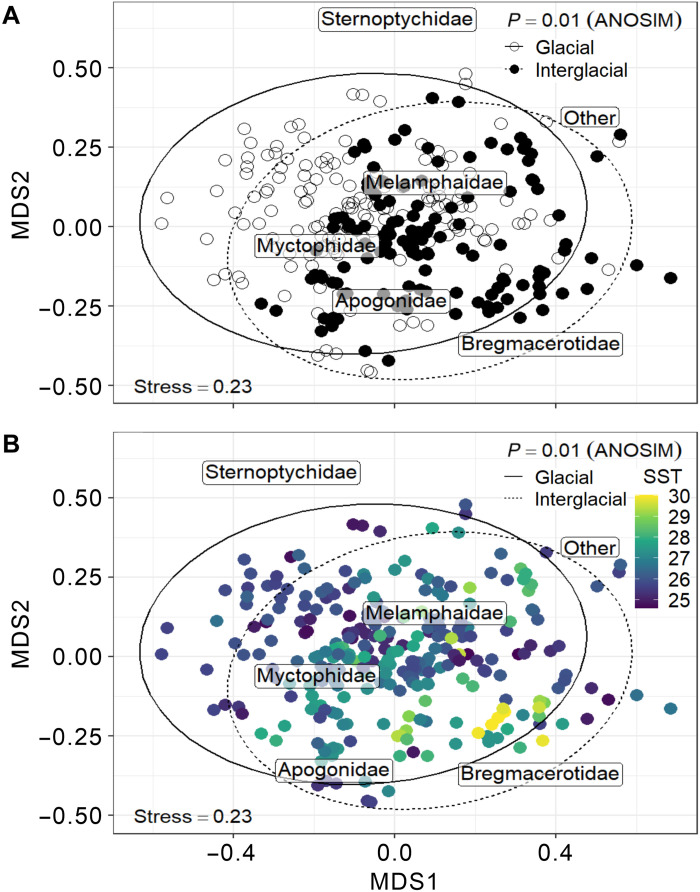
Nonmetric multiple dimension scaling on species OARs over the past 460 ka. Species OARs were calculated using a 10-ka moving window (i.e., the same data used for estimating Hill numbers) and then converted to Hellinger distance (*59*) for nonmetric multiple dimension scaling (nMDS). The text labels show the weighted averages (by OARs) of ordination scores of the five most abundant taxa. The symbol and oval colors in (**A**) indicate the glacial (i.e., open) and interglacial (i.e., closed) periods. The color key in (**B**) shows the mean SST in moving windows. The analysis of similarities test indicates the taxonomic composition was significantly different between glacial and interglacial periods.

Our results show a sharp decrease of OAR and Hill numbers following the termination of warming phases during past warmer-than-present interglacial periods (MISs 5e and 11c) (Fig. 2, A and B), indicating declining fish production and diversity subsequently after the peak warmth. We further identified fish composition shifting among cool, warm, and super warm pelagic ecosystems. Members of *Ceratoscopelus*, *Diaphus*, and *Lampanyctus* (Myctophidae) are broad thermal tolerant taxa that occur throughout the record (Figs. 1 and 5), while the small pelagic codlets (Bregmacerotidae) could tolerate super warm conditions during MISs 5e and 11c (Fig. 5).

## DISCUSSION

The otolith record at site ODP 1115B mirrors the general mesopelagic fish community in an open-ocean setting (*13*–*15*, Fig. 1), similar to otolith assemblages reported by Jones and Checkley Jr. (*12*) from the Santa Barbara Basin. Carbonate preservation in the Solomon Sea is excellent, and no notable changes across G-IG transitions were found by a previous study (*28*). The high sedimentation rate (*29*) and otolith flux compared to other pelagic sites (*12*) have likely enabled a rapid burial and thus enhanced the preservation of fish otoliths at site ODP 1115B. In addition, aragonitic otoliths could rapidly shift to calcite in their early diagenesis (*14*), which prevents further dissolution of the otoliths (*30*). The present continuous otolith record, which remarkably contains information on both fish production and species diversity, thus provides an exceptional way to evaluate the possible range of fish community sensitivities in response to natural climate change over geological time scale. Our data demonstrate that the mesopelagic fish production was higher during interglacial periods and lower during the glacial periods at least at the southwestern margin of the Pacific Warm Pool (Fig. 2A). By contrast, the species diversity shows an opposite pattern with lower (higher) diversity during interglacial (glacial) periods (Fig. 2B and fig. S5).

According to modern observations (*8*), marine productivity and seawater temperature are the major controlling factors of mesopelagic fish community. Both productivity and temperature fundamentally influence feeding activity through individual metabolic rate and food source acquisition. On the G-IG scale, it is generally thought that tropical marine productivity is higher during the glacial periods than that during the interglacial periods (*31*). However, biogenic carbonate–derived SAR and TOC (figs. S7 and S8) indicate a rather different productivity evolution at our study site. These data also do not show a strong correlation with OAR, suggesting that the OAR is likely independent of local productivity.

**Fig. 6.**
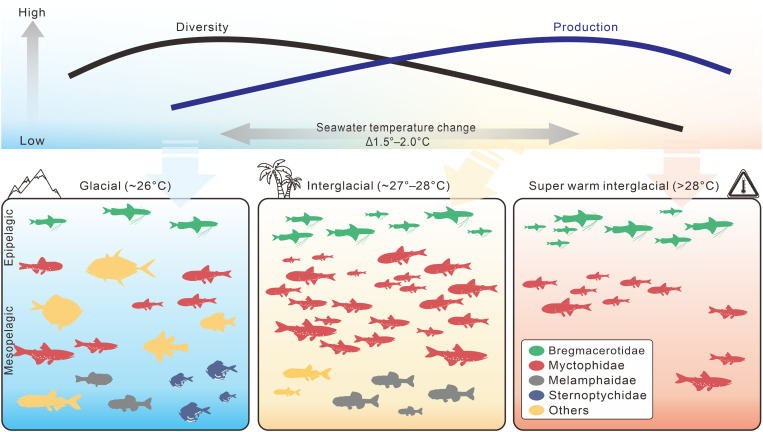
Dynamic patterns in mesopelagic fish production and diversity driven by seawater temperatures. Fish production shows lower (higher) values during glacial (interglacial) periods, whereas the diversity exhibits the opposite trend. These patterns correlate with hump-shaped gradients of seawater temperatures, with the diversity tipping point occurring at lower temperatures than that of the production by approximately 1.5° to 2.0°C. Furthermore, during warmer-than-present interglacial periods when the temperature exceeds the higher tipping point, both the production and diversity decline sharply after the maximum warmth is attained, highlighting the sensitivity of the mesopelagic fish community to a warmer ocean.

On the contrary, the SST and UTT records from site MD05-2925 show temporal patterns similar to those of OAR (Fig. 2, A, C, and D). We note that the tipping point seawater temperatures for fish diversity are lower than that for production by ~1.5° to 2.0°C (Fig. 4 and fig. S11). Fish diversity peaks when the production is still rising during glacial periods, whereas diversity already declines when the production reaches the maximum during interglacial periods with higher temperatures (Fig. 4, B and C). The fish diversity and production subsequently peak at different temperatures, forming two different hump-shaped temperature gradients (Fig. 6). Therefore, the oscillations of the mesopelagic fish diversity and production at G-IG time scale (Fig. 2, A and B) likely result from this two-hump-shaped temperature gradient model (Fig. 6).

Our otolith record may be explained by the intermediate disturbance hypothesis that predicts that the highest diversity occurs at levels of moderate disturbance (*32*). Temperatures may be more variable during the glacial periods (*33*), creating an environmental disturbance at intermediate intensity and frequency that allows more species (opportunists and competitors) to coexist. By contrast, a more stable community may exist in favor of a few successful competitors during the interglacial periods, with less frequency and amplitude of disturbance. However, the level of intermediate disturbance is challenging to constrain. Alternatively, there is accumulating evidence supporting the hypothesis that marine biodiversity may decline in a warmer ocean (*34*–*37*). Species tend to occupy a narrow thermal niche, at warmer temperatures only the broadly temperature-tolerant species can exist. However, at certain temperatures (near the thermal tipping point for diversity), there are higher levels of diversity because both warm-adapted and cool-adapted species can coexist.

Modeling and survey studies have indicated a negative impact of temperature on fish abundance and diversity (*38*, *39*). However, these studies are often based on a very short time scale, i.e., “snapshot”; thus, it remains unclear whether these findings are applicable to a longer time scale. An independent modeling study using fish fossils suggested a marked increase in pelagic fish biomass during the Early Eocene Climate Optimum due to a substantial improvement in trophic transfer efficiency (*40*). Although the mechanism for changes in trophic efficiency was not explicit, our otolith record does suggest higher mesopelagic fish production during warmer periods (Fig. 2A). The relatively simplified interglacial communities could facilitate a more efficient, shorter energetic transfer pathway due to less energy used in biotic interactions, such as for resource competition and predation avoidance.

During MISs 5e and 11c when the local seawater temperatures exceeded the Holocene level (Fig. 2, C and D), both fish production and diversity subsequently declined (Fig. 2, A and B, and fig. S5). This shift deviates from the general G-IG pattern but can still be explained by our hump-shaped temperature gradient models. This decline may be attributable to a low oxygen level in the seawater following a prolonged period of decreasing oxygen solubility as the ocean warms. Hypoxia mortality can occur even when oxygen concentration exceeds 6 mg/liter at 30°C (*41*). Our data reveal that the extremely high temperature during MISs 5e and 11c may have an extensive impact on the mesopelagic fish community at the southwestern margin of the Pacific Warm Pool and possibly also communities in other hydrographically similar regions, providing a critical piece of evidence on how fish production and diversity in this region may change in a warmer future. Higher ocean temperatures likely sustain and favor the broadly temperature-tolerant small mesopelagic fishes (*17*). The worldwide distributed, almost ubiquitous myctophids and bregmacerotids might be some of the few fish in the region that thrive and propagate to large numbers in the forthcoming warming condition (Fig. 5).

In the Humboldt Current system, regime shifts of epipelagic fishes like anchovies and sardines are of particular research interest due to the high economic value of the fish (*42*). Instead of today’s typical epipelagic anchovies, a scenario in which more mesopelagic fish occupy the east Pacific upwelling ecosystem in warmer periods has been demonstrated by fish fossil records (*17*). Similarly, our results also indicate a higher mesopelagic fish production during warmer interglacial periods, despite the absence of upwelling at our study site. Our G-IG records further add to this finding, showing that, at least at the southwestern margin of the Pacific Warm Pool, the increase in mesopelagic fish production during warm periods is not only limited to interglacial periods but can be also observed when comparing glacial and interglacial periods.

Another interesting finding from our data is a pronounced decreasing/increasing secular trend in the OAR/Hill numbers over the past 460 ka (Fig. 2, A and B), suggesting continuous dwindling fish production and growing diversity in the past four glacial cycles. Notably, including present-day and Holocene conditions (<10 ka ago), the peak production is substantially less than that of the MISs 7, 9, and 11 (Fig. 2A). There is currently no explanation for this observation, but it is not biased by otolith preservation (*43*), as older OAR is higher than the younger ones. Further studies on fish records on a much longer geological time scale may shed new light on these trends. Our otolith record is nearly independent of anthropogenic disturbance and human-induced global warming, except for the last one or two subsamples. Therefore, this record can be regarded as critical ecological baseline data, with clear and urgent implications for fishery management associated with pelagic and deep-sea ecosystems. Future work on longer geological time scales and broader geographical sampling coverage are expected to reveal a more comprehensive view of the past mesopelagic fish community.

## MATERIALS AND METHODS

### Summary of the sediment core

ODP Hole 1115B is located in the Solomon Sea (9°11′S, 151°34′E; water depth, 1149 m), which is at the seasonal southern margin of the Pacific Warm Pool (*25*, *44*). The thermocline waters here are sourced from Subarctic Mode Water and Antarctic Intermediate Water (*25*, *45*). Thus, the thermal and hydrological conditions at the surface ocean and thermocline in the Solomon Sea are sensitive to both high- and low-latitude climatic perturbations. The total length of ODP 1115B core is 216.4 m, and the sediment composition consisted mainly of clay to silty clay (*29*). The preservation of carbonate fossils in this basin is good to excellent without clear G-IG variations in dissolution conditions, according to observations from a nearby site MD05-2925 (*26*) (9°15′S, 151°34′E; water depth, 1642 m; fig. S9D). Calcareous fossils and subfossils including fish otoliths are extraordinarily abundant in this core (*19*). Details of the age model are published (*29*), which is based on paleomagnetic data, biostratigraphy, as well as stable oxygen isotopes of planktonic foraminifera, *Trilobatus sacculifer* (300 to 355 μm). Sea surface and UTT data are from Lo *et al.* (*25*, *26*), based on the temperature calibration of Anand *et al.* (*46*) multispecies composite equation.

### Otolith preparation and identification

Otoliths constituted the major fish remains and only a single fish tooth and two vertebrae were found in the core. The otoliths were extracted from each of the subsamples (10 to 15 cm^3^ of sediment with 15- to 25-g dry weight) via wet sieving through a 500-μm mesh, which is a common mesh size for processing otoliths (*13*–*15*). The average sampling interval is 5 to 10 cm along the core, and the temporal resolution is around 1 to 4 ka per sample. A total of 343 subsamples were available, of which 51 did not contain any otoliths (data S1). All otoliths (sagittal otoliths) within residues larger than 500 μm were picked, counted, and identified using our reference collections (*47*) and published literature (*48*–*50*). When similar-sized left or right otoliths of the same taxon were present in a single subsample, they were counted only once to avoid overestimation from the same individual. The otoliths of mesopelagic fishes, both extant species (*9*, *10*) and their fossil records (*11*, *51*), are well known at the global scale at least at the generic level and thus can be identified with confidence to genus or even to species by specialists (*9*, *10*). However, because of the large number of juvenile otoliths that lack characteristic features (*13*–*15*), our identifications are conservative and most specimens were recognized to genus or above. The genus-level taxonomic resolution was used to accommodate the largest number of otoliths possible and throughout our diversity analyses, which we note is common for paleoecological practice (*13*–*15*). Examples of each identified otolith taxon are presented in figs. S12 to S17.

### Accumulation rates and productivity proxies

The OAR is calculated as follows: OAR (ind cm^−2^ ka^−1^) = number of otoliths (ind g^−1^) × LSR (cm ka^−1^) × DBD (g cm^−3^) (*52*); where LSR is the linear sedimentation rate (cm ka^−1^) and DBD is the dry bulk density (g cm^−3^) (*19*). Similarly, the SAR is as follows: SAR (g cm^−2^ ka^−1^) = SF (>63-μm coarse sand fraction weight/total weight of sample) × LSR (cm ka^−1^) × DBD (g cm^−3^) (*52*). The weight of otoliths is excluded from the calculation of SAR. The otolith weights do not exceed 1.26% of the weight of coarse sand in each sample, indicating that our SAR is independent of otolith weight (data S3). Here, the SAR is used as a proxy for productivity (*24*, *53*) based on two prerequisites: (i) predominance of biogenic origin in the coarse sand fraction and (ii) limited carbonate dissolution and bottom-water currents (*54*). The biogenic calcium carbonates in the upper 100 m (including all our samples here) of the core ODP 1115B exceed 60% of the total dry weight content (*19*), and the good preservation of foraminiferal shells suggest limited carbonate dissolution (*29*). TOC is measured by the element analyzer of soil TOC cube (ELEMENTAR). Freeze-dried and homogenized sediment (~50 mg) was heated up stepwise from room temperature to 600°C to extract the organic component. Reproducibility is determined by replicated measurements of commercial standard LECO soil standard (total carbon 0.726 ± 0.016%, *n* = 105). TOC mass accumulation rate is calculated as the method mentioned above.

### Time series analyses and smoothing

Spectral analyses were conducted using ARAND software (*55*). Time series for single-spectrum analysis was resampled to 4-ka resolution. Insolation and orbital parameters, at 1-ka step, were based on the La2004 solution (*56*). We applied a 10-ka moving window to smooth our original data. Each time point thus contains an averaged value from the plus and minus 5-ka original data, and thus, the moving window of the first and last 5-ka were truncated. The 10-ka moving window was chosen on the basis of its ability to capture the most features compared to other time windows (versus 0- to 20-ka moving windows; fig. S18), thereby allowing the inclusion of a high number of taxa with a median sample size over the past 460 ka. Furthermore, the original data are based on a total of 343 sediment slices and each slice contain 7 ± 1.9 (SD) otoliths and 3 ± 1.7 species (or taxa). It is potentially problematic to estimate the alpha diversity in each time snapshot (sediment slice) with such a small sample size. This 10-ka moving window technique allows us to better estimate the gamma diversity over a longer time period at each time slice. For each moving window, we are able to increase the number of otoliths per sample to 27 ± 10.8 otoliths and 9 ± 2.5 species (or taxa), which is a more appropriate sample size to estimate OAR and species diversity (see below).

### Species diversity analysis

We calculated the species diversity within a 10-ka moving window (see above) using Hill numbers (*21*) or the effective numbers of equally abundant species in a hypothetical assemblage (*57*). Hill numbers (*21*) are generally regarded as the choice of diversity measure in ecology (*58*) despite being relatively underused in paleo communities. The Hill numbers obey the “doubling property” (i.e., diversity doubles when two identically distributed but distinct communities combine), so they are ecologically intuitive with similar mathematical properties to species richness (*57*). The Hill number, *^q^D* = (∑*p_i_^q^*)^(1/(1-*q*))^, is a function of the *q*th power sum of the relative species abundance (*p_i_*). Thus, the order *q* controls the sensitivity of Hill numbers to the relative species abundance. For example, when *q* = 0, ^0^*D* weights all species equally, and the Hill number reduces to the species richness. For *q* ≍ 1, ^1^*D* weights species in proportion to their abundance and can be interpreted as the effective number of abundant species. For *q* = 2, ^2^*D* measures the effective number of highly abundant or dominant species. The Hill numbers of order *q* = 0, 1, 2 are mathematically equivalent to the species richness, the exponential of Shannon entropy, and the inverse of Simpson concentration index (*23*), respectively. Following Chao *et al.* (*23*), we adopted these three most commonly used diversity indices and referred to them as species richness (^0^*D*), Shannon diversity (^1^*D*), and Simpson diversity (^2^*D*). The observed diversity (or Hill numbers) depends on sample completeness or coverage, which is the ratio between the number of individuals represented by the species present in a sample and the actual number of individuals in an assemblage. The sample coverage can be estimated from the proportion of singletons and doubletons in the samples (*22*). Therefore, the observed Hill numbers were rarefied or extrapolated to generate a smooth diversity accumulation curve using bootstrap resampling (repeating 1000 times) for each 10-ka moving window (fig. S3). We then extracted the Hill numbers at a fixed sample coverage (i.e., 85% completeness) to make fair diversity comparisons (*22*, *23*). See details of the calculation of the Hill numbers in Supplementary Text. The species OARs were also converted to Hellinger distance (*59*) and subjected to nonmetric multiple dimension scaling (nMDS). We overlaid the weighted averages (by OARs) ordination scores of the five most abundant species on the nMDS plot. We used the analysis of similarities (ANOSIM) to examine the composition difference between the G-IG periods. We used the R package vegan to compute nMDS and ANOSIM.

### Generalized additive modeling

We used GAM to examine the secular trends of OAR and diversity (^1^*D*, ^2^*D*, and ^3^*D*) while considering the nonlinear effects of sea surface (or upper thermocline) temperature and temporal autocorrelation (i.e., by adding residual correlation structure corAR1). The GAMs were modeled with a Gaussian distribution, an identity link function, and a cubic regression spline. We used a low smoothing parameter (*k* = 6) on time to reveal long-term trends and automatic selection of smoothing parameters on temperature to identify potential thermal threshold. All GAM models (OAR, ^1^*D*, ^2^*D*, and ^3^*D*) are significant smooth functions of time and seawater temperatures. The GAM models were fitted using the R package mgcv and validated by the residuals versus fitted values, QQ plot, and partial autocorrelations function.

## Supplementary Materials

### This PDF file includes:

Supplementary Text Figs. S1 to S18 Legends for data S1 to S3 References
