# Supplementary material for: Ocean temperature drove changes in the mesopelagic fish community at the edge of the Pacific Warm Pool over the past 460,000 years
Source: Sci Adv. 2023 Jul 7;9(27):eadf0656. doi: 10.1126/sciadv.adf0656 (PMC10328404; doi:10.1126/sciadv.adf0656)
Supplement: Supplementary file 1 — Supplementary Text Figs. S1 to S18 Legends for data S1 to S3 References [file sciadv.adf0656_sm.pdf]

Science Advances
AAAS

Supplementary Materials for

Ocean temperature drove changes in the mesopelagic fish community at the edge of the Pacific Warm Pool over the past 460,000 years

Chien-Hsiang Lin *et al.*

Corresponding author: Chien-Hsiang Lin, chlin.otolith@gmail.com; Li Lo, lilo115@ntu.edu.tw

Sci. Adv. **9**, eadf0656 (2023)
DOI: 10.1126/sciadv.adf0656

The PDF file includes:

Supplementary Text
Figs. S1 to S18
Legends for data S1 to S3
References

Other Supplementary Material for this manuscript includes the following:

Data S1 to S3

Supplementary Text

A step-by-step procedure for calculating the Hill numbers

Any diversity estimates are affected by sample sizes (or sampling efforts) and the abundance in each sample. This is especially problematic when using traditional diversity indices, such as species richness and Shannon Entropy. These indices may underestimate diversity from samples with low abundance, hindering the diversity comparison across spatial and temporal gradient. Therefore, we used a resampling approach to rarefy or extrapolate the Hill number to a fixed sample coverage (or a fixed sampling effort) (*22*). In plain language, we first resampled the otoliths from one individual to 2 times the total number of individuals in a 10-ka moving window to construct a Hill number accumulation curve.

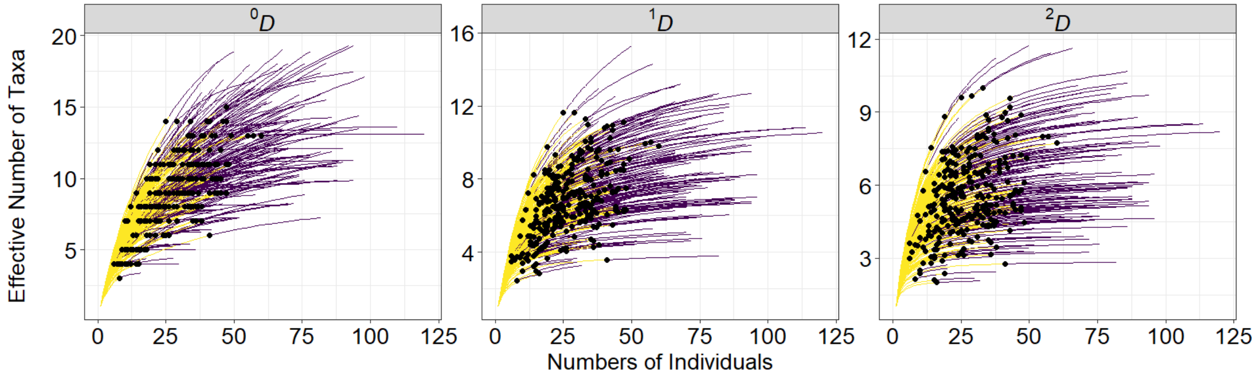

Fig. S1. Hill number (effective number of taxa) accumulation curve based on resampling the number of individuals (number of otoliths) from the ODP Hole 1115B.

As shown in fig. S1, the Hill numbers (or effective number of taxa, y-axis) increase with the number of resampled otoliths (x-axis). Here, each curve represents a 10-ka moving window. The yellow line is the rarefied part of the curve, the purple line is the extrapolated part, and the symbol is the observed Hill number. Next, we calculate the sample coverage for each resampled number of otoliths based on the proportion of singleton and doubleton species (i.e., species represented by one or two otoliths) (*22*).

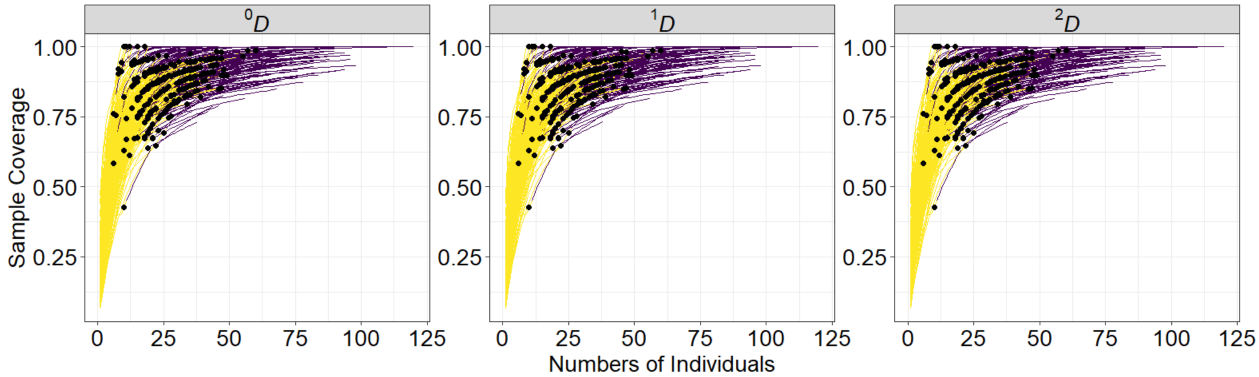

Fig. S2. Relationship between sample coverage and number of individuals (number of otoliths) from the ODP Hole 1115B.

In fig. S2, the sample coverage on the y-axis is the ratio between the number of otoliths represented by the species in a 10-ka moving window and the actual number of individuals in an assemblage (i.e., the ratio between estimated and true diversity). Even if we fixed the number of otoliths on the x-axis to standardize the diversity (i.e., Hill numbers), for example based on 50

randomly selected otoliths on the x-axis, the diversity estimates remain varied as shown on the y-axis based on the sample coverages (or sampling efforts). Therefore, standardization of all Hill number estimates to a fixed sample coverage (i.e., 85%, vertical dashed line) to fairly compare the diversity in each moving window is necessary (fig. S3).

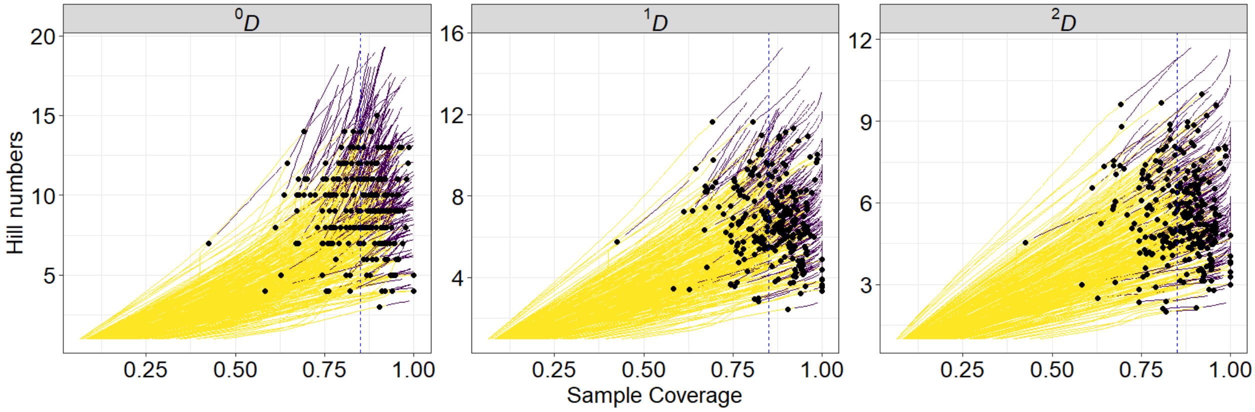

Fig. S3. Coverage-based diversity accumulation curves based on Hill numbers of order = 0 (^0D, species richness), 1 (^1D, Shannon diversity), and 2 (^2D, Simpson diversity). The yellow lines indicate the rarefied (or interpolated) sample coverage, and the purple lines indicate extrapolated accumulation curves based on 1000 permutations. Closed circles indicate the observed Hill numbers. Because not all accumulation curves have reached their respective asymptote, we choose the sample coverage = 85% to standardize the Hill number estimates. Note that ~7.2% of moving windows has lower sample coverage (< 85%), and the lowest maximum sample coverage among moving windows is 62.8%. In other words, the Hill numbers in ~7.2% of moving windows could be underestimated, but the Hill numbers in most moving windows are standardized to equal sample coverage (or sampling efforts) of 85%.

This process was repeated 1000 times to generate mean Hill number accumulation curves in figs. S1-S3. This step-by-step procedure for calculating the Hill numbers has demonstrated that we tackled the sample size/sampling effort issues in estimating diversity, which is always challenging. To our knowledge, rarefaction and extrapolation with Hill numbers (*23*) is currently the best tool for unbiased diversity estimates. It was developed to tackle the uneven sample size issue and has become the standard protocol for diversity estimates in ecological studies.

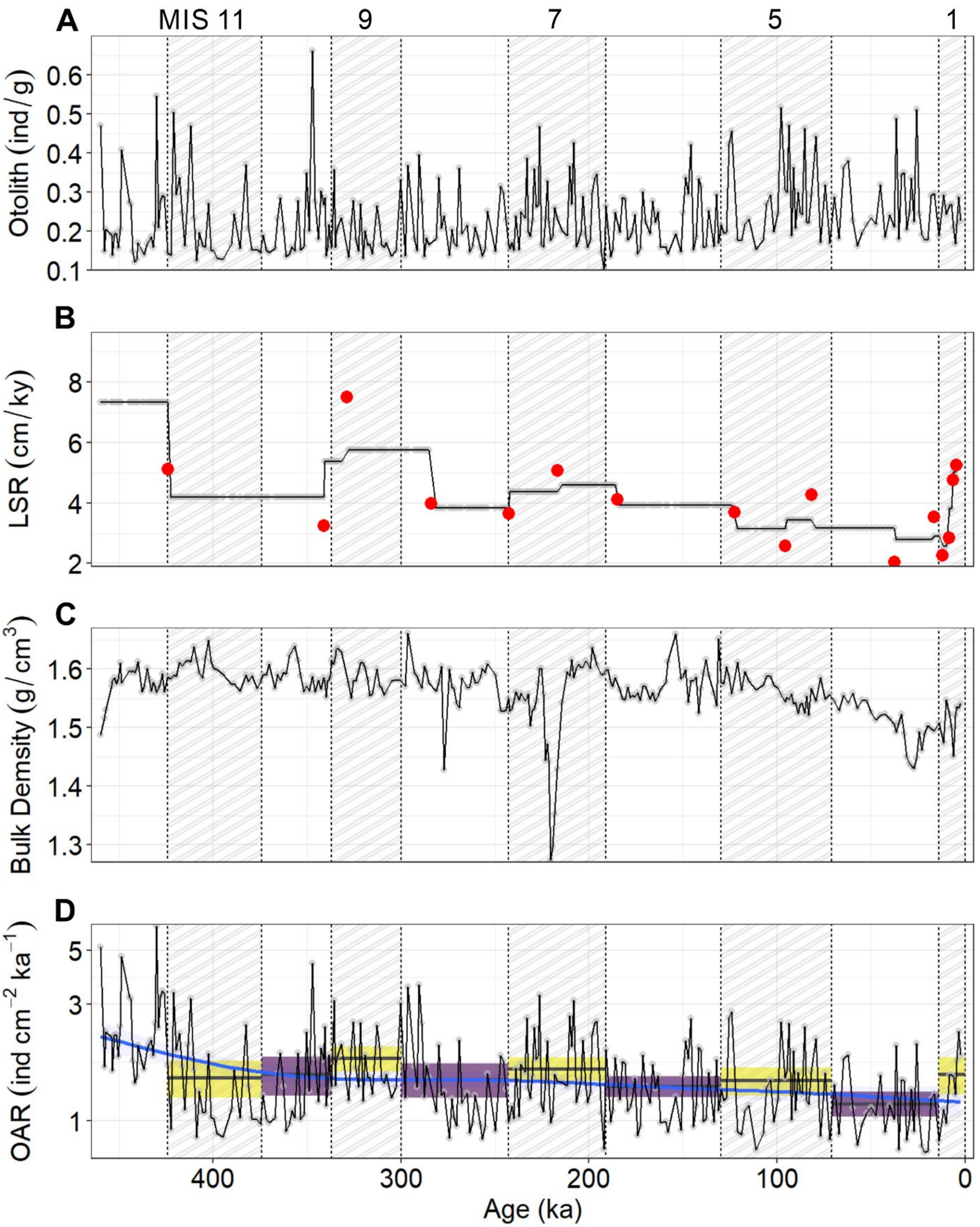

Fig. S4. Raw otolith abundance (A), linear sedimentation rate (LSR, B), sediment bulk density (C), and otolith accumulation rate (OAR, D) over the past 460 ka. Hatched rectangles indicate interglacial periods (Marine Isotope Stages 1, 5, 7, 9, 11 on the upper x-axis). The red circles in panel (**B**) indicate the original LSR data and the black line indicates the averaged value between any two red circles. The black horizontal lines in panel (**D)** indicate the mean and the shaded areas show the 95% confidence intervals, and the blue line ($p < 0.05$) and shaded areas show generalized additive model (GAM) fits on smoothed OAR using a moving-window approach with 95% confidence intervals.

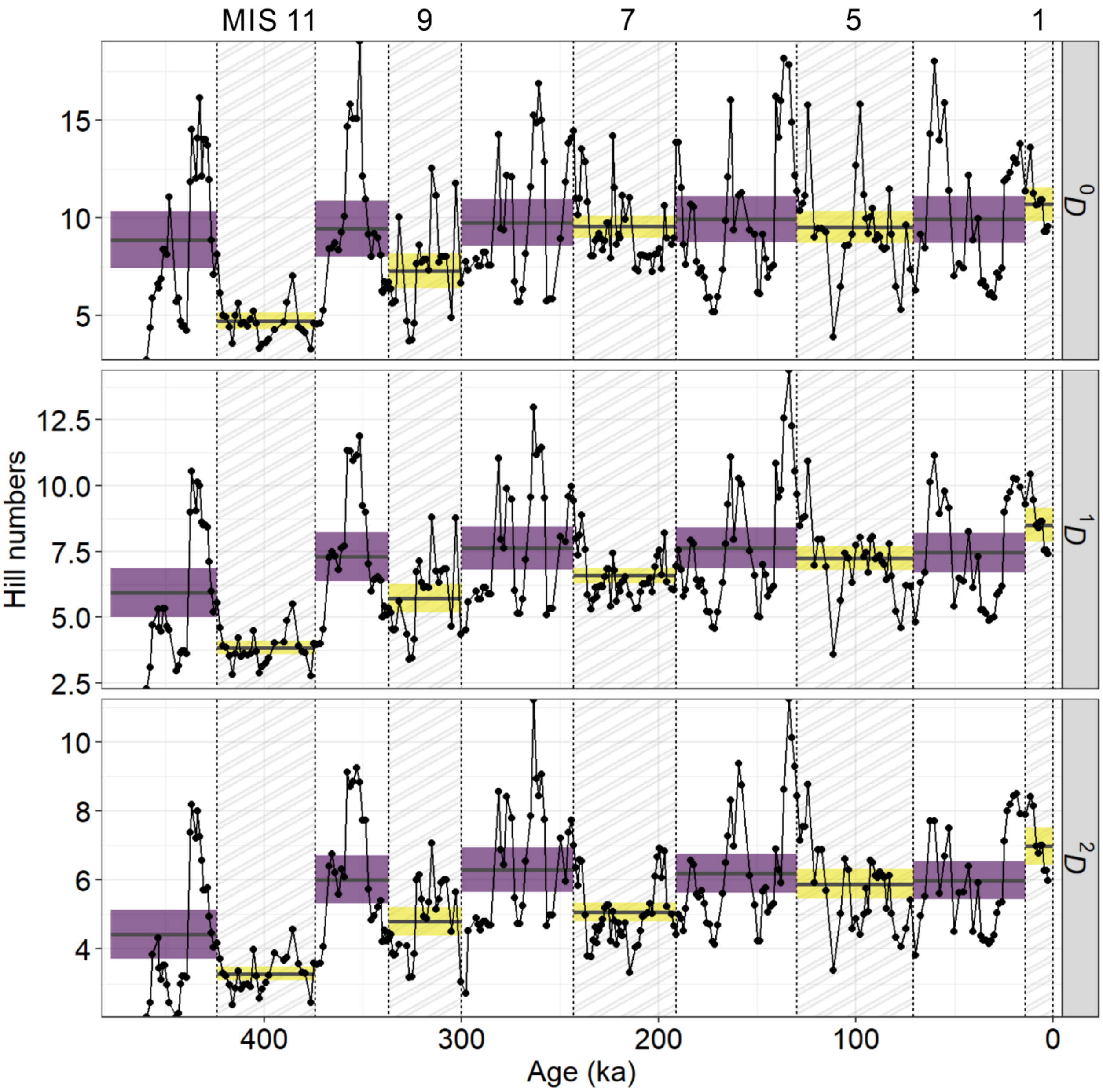

Fig. S5. Hill numbers (^qD) of order $q = 0$ (^0D), $q = 1$ (^1D) and $q = 2$ (^2D) based on 85% sample coverage over the past 460 ka. Hatched rectangles indicate interglacial periods (Marine Isotope Stages 1, 5, 7, 9, 11 on the upper x-axis). The black horizontal lines show the mean and the shaded areas show the 95% confidence intervals.

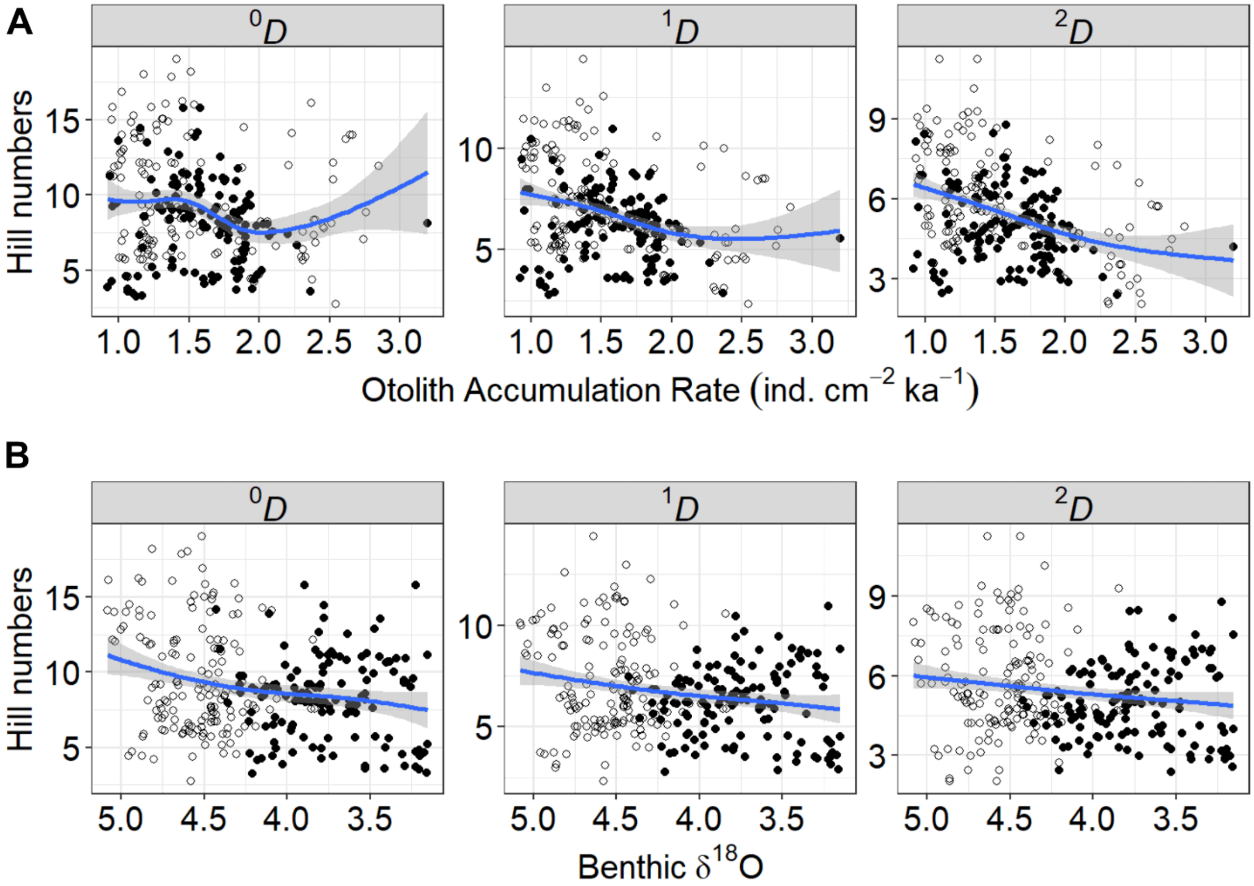

Fig. S6. Hill numbers (^{q}D) of order q = 0 (^{0}D), q =1 (^{1}D) and q = 2 (^{2}D) based on 85% sample coverage against (A) otolith accumulation rate and (B) benthic δ^{18}O. Only significant trend lines are shown ($p < 0.05$). Open symbols = glacial periods and closed symbols = interglacial periods. Otolith diversity decreases with otolith accumulation rate, but increases with benthic δ^{18}O. The blue line and shaded areas show generalized additive model (GAM) fits with 95% confidence intervals. The decline of Hill numbers and the explanatory power (adjust-R^2) of GAM are the greatest for ^{2}D, followed by ^{1}D and ^{0}D, suggesting that OAR exerted stronger control on the diversity of dominant (^{2}D) and abundant (^{1}D) species (i.e., myctophids) (**A**). Moreover, the slopes of GAM fit between diversity and δ^{18}O decrease from ^{2}D, ^{1}D to ^{0}D, suggesting that glacial-interglacial change had less impact on the dominant (^{2}D) and abundant (^{1}D) species (i.e., myctophids) (**B**).

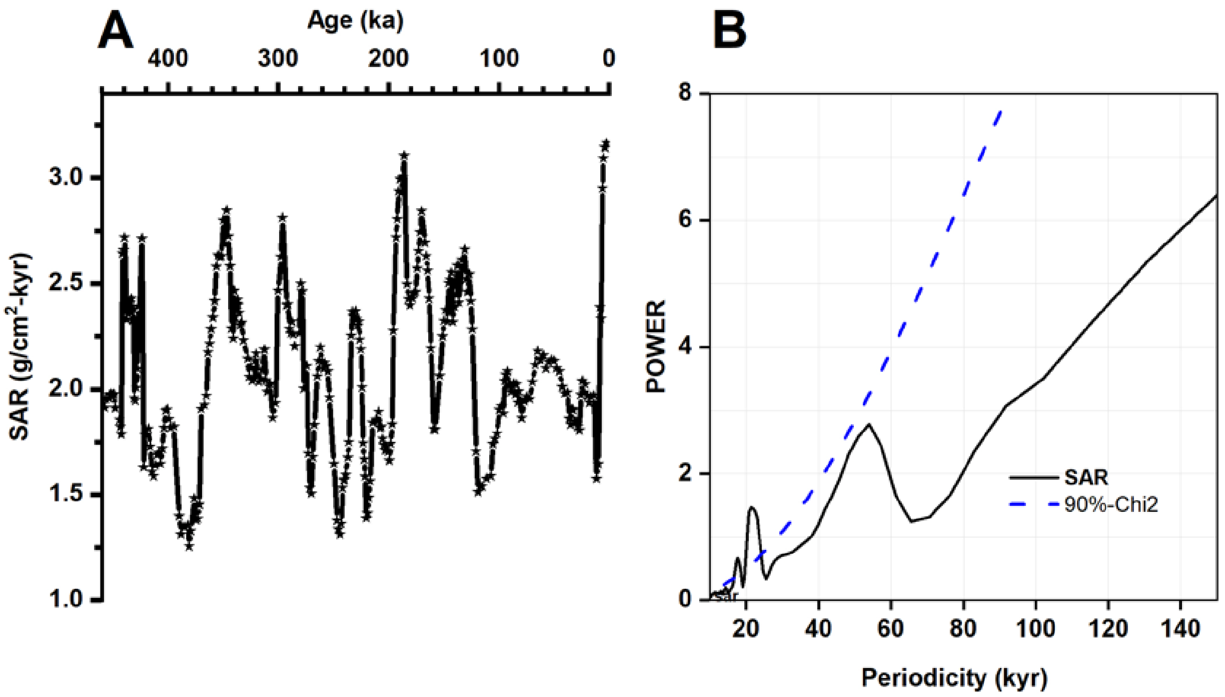

Fig. S7. Sand accumulation rate (SAR, A) of ODP Hole 1115B and power spectrum for SAR (B). SAR is calculated using a 10-ka moving window (Materials and Methods) and the blue dashed line indicates the 90% confidence line (*60*).

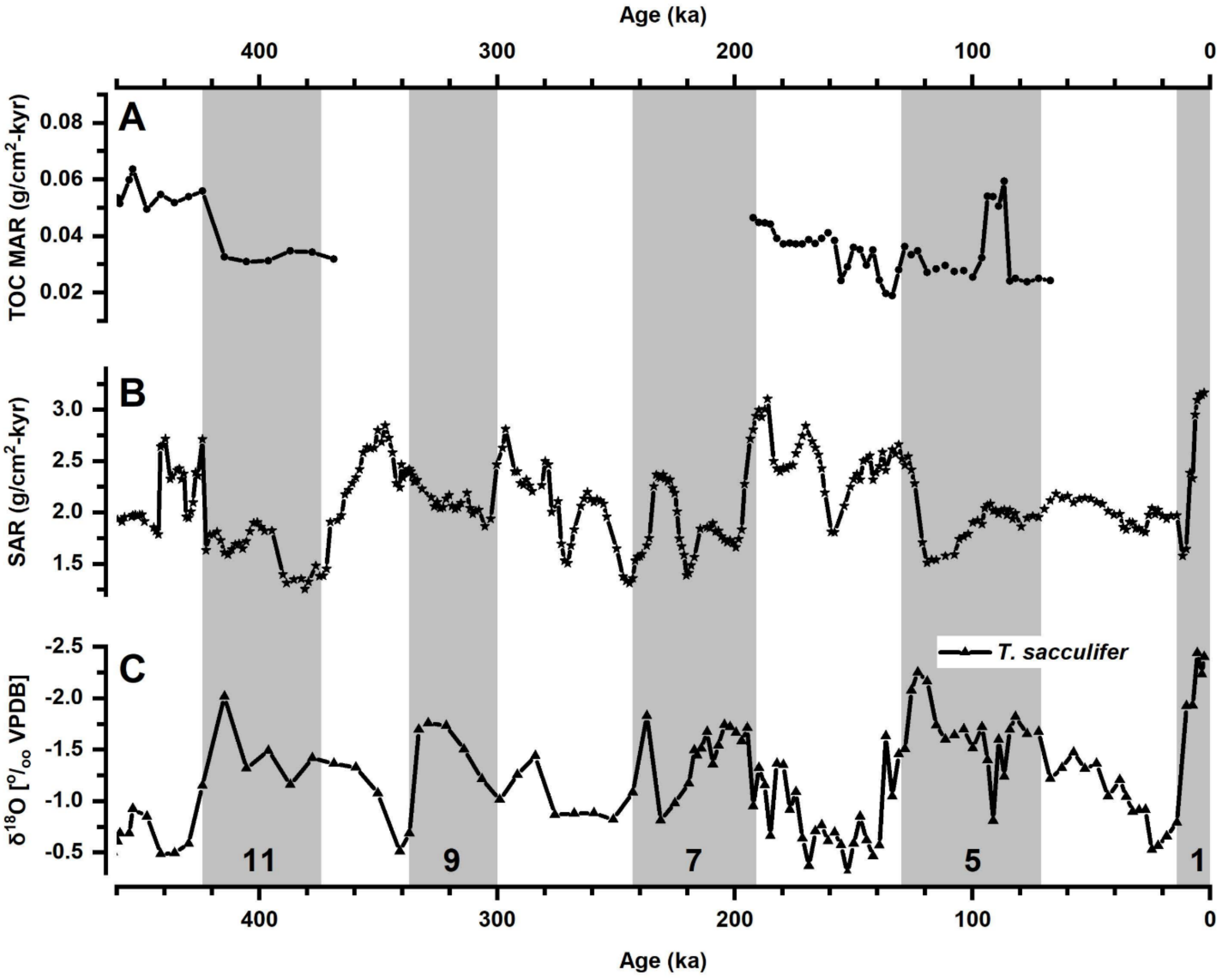

Fig. S8. Total organic carbon mass accumulation rate (TOC MAR, A), sand accumulation rate (SAR, B) and planktonic foraminiferal oxygen isotope (C) from the ODP Hole 1115B. Accumulation rate is calculated using a 10-ka moving window (Materials and Methods) and the gray bars represent interglacial periods (Marine Isotope Stages 1, 5, 7, 9, 11).

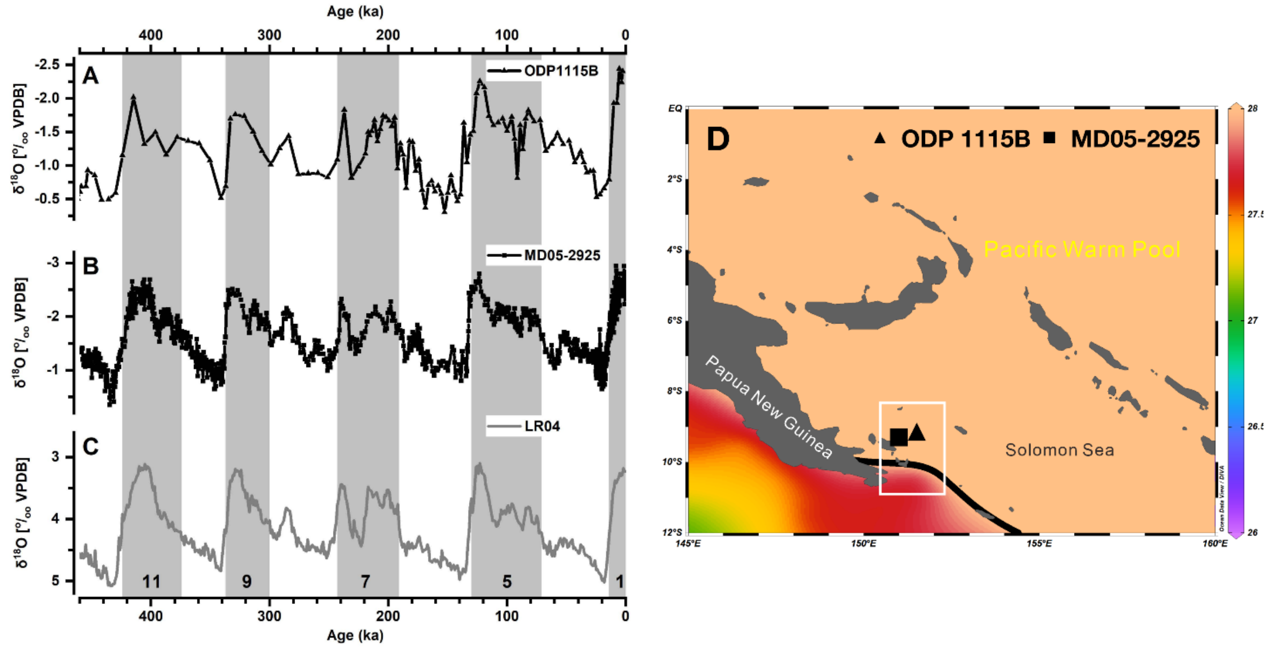

Fig. S9. Planktonic foraminiferal oxygen isotope records of ODP Hole 1115B (A) and MD05-2925 (B) against the global composite benthic foraminiferal stratigraphy (C) (*27*), and locations of ODP Hole 1115B and MD05-2925 plotted with annual mean sea surface temperature from World Ocean Atlas (D). Oxygen isotopic composition of surface-dwelling planktonic foraminifera *Trilobatus sacculifer* and *Globigerinoides ruber* from ODP Hole 1115B (**A**) and MD05-2925 (**B**) were previously reported by Chuang et al. (*29*) and Lo et al. (*26*), respectively. Mean sea surface temperature data are from Locarnini et al. (*61*). The gray bars in (**A**), (**B**) and (**C**) represent interglacial periods (Marine Isotope Stages 1, 5, 7, 9, 11). The boundary of the Pacific Warm Pool is delineated by the black line in (**D**).

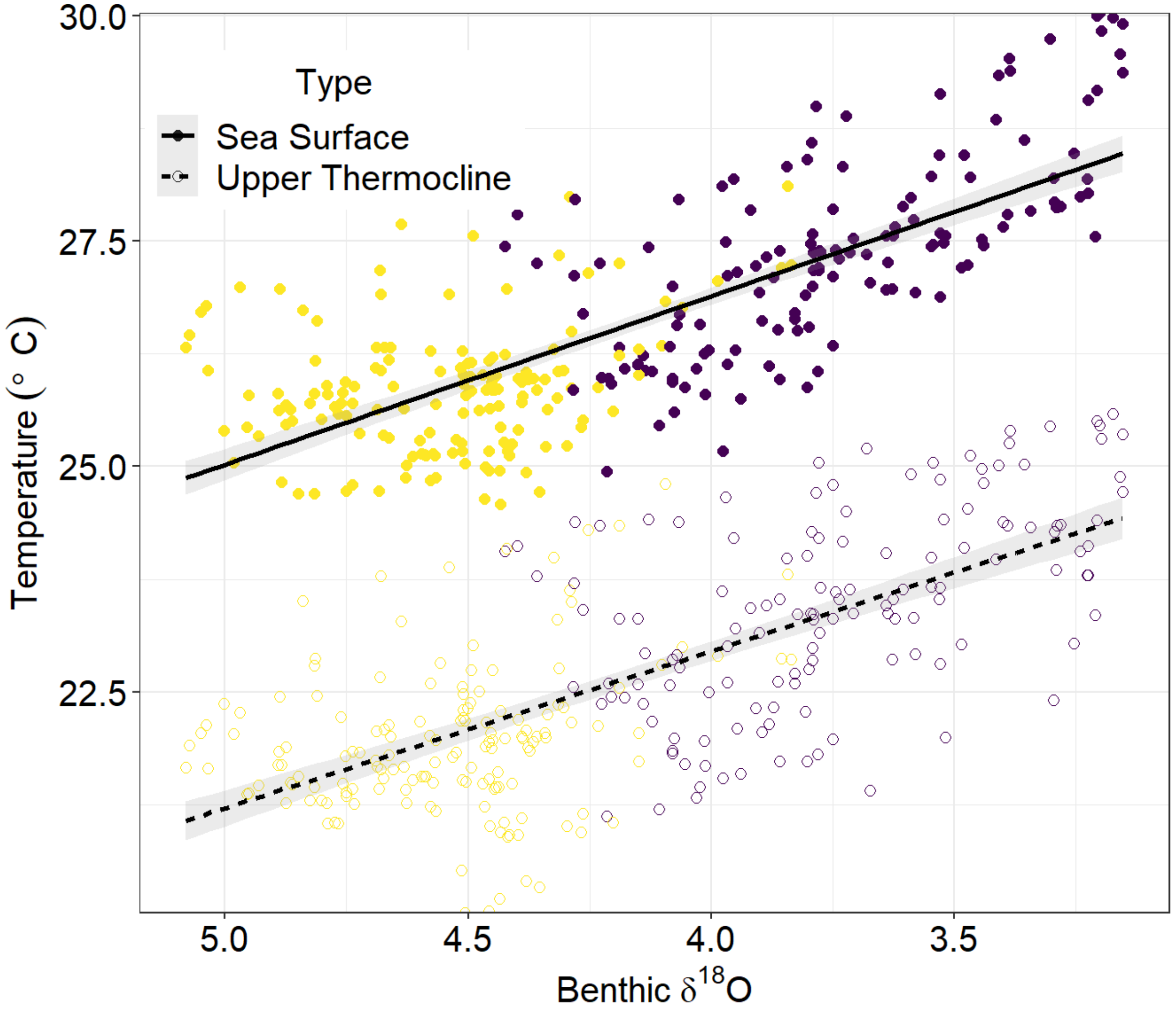

Fig. S10. Sea surface/upper thermocline temperature as a function of benthic δ^{18}O. Only significant trend lines are shown ($p < 0.05$). Sea surface temperature and upper thermocline temperature data are from core MD05-2925 (Materials and Methods). Yellow symbols = data from glacial periods and purple symbols = data from interglacial periods.

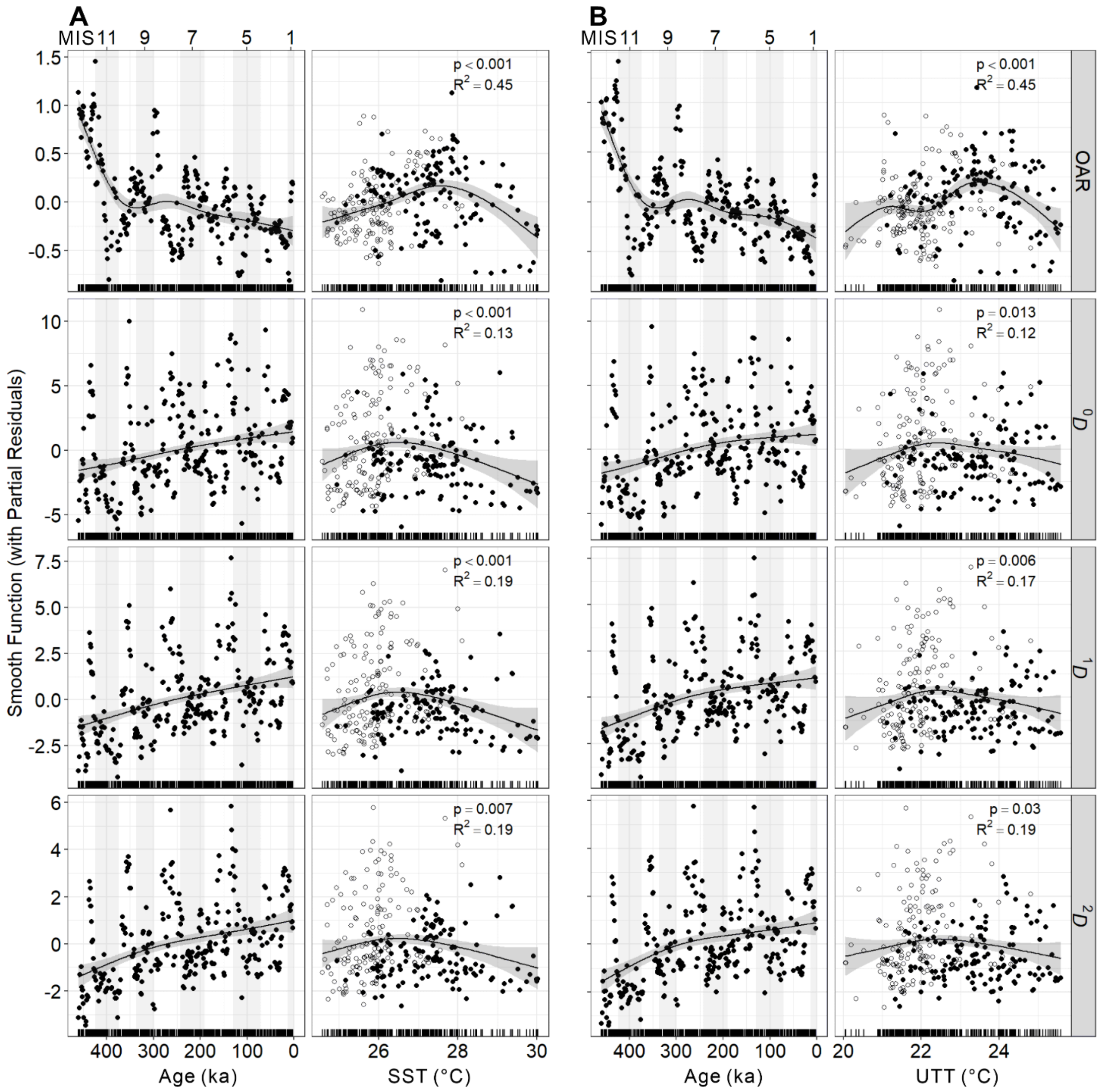

Fig. S11. Response curves from the generalized additive models (GAM) based on otolith accumulation rate (OAR) and Hill numbers of order = 0 (^0D, species richness), 1 (^1D, Shannon diversity), and 2 (^2D, Simpson diversity). Vertical panel **A** shows the GAM response curves (black lines ($p < 0.05$) with gray shades as 95% confidence intervals) as functions of time (left) and the mean sea surface temperature (SST, right) in 10-ka moving windows. Vertical panel **B** shows the response curves as functions of time (left) and the mean upper thermocline temperature (UTT, right) in 10-ka moving windows. Symbols indicate the partial residuals of the GAM fits. Gray rectangles on the left sub-panels indicate interglacial periods (Marine Isotope Stages 1, 5, 7, 9, 11 on the upper x-axis). The open symbols on the right sub-panels indicate glacial periods, while closed symbols show interglacial periods. The rug plots indicate data density.

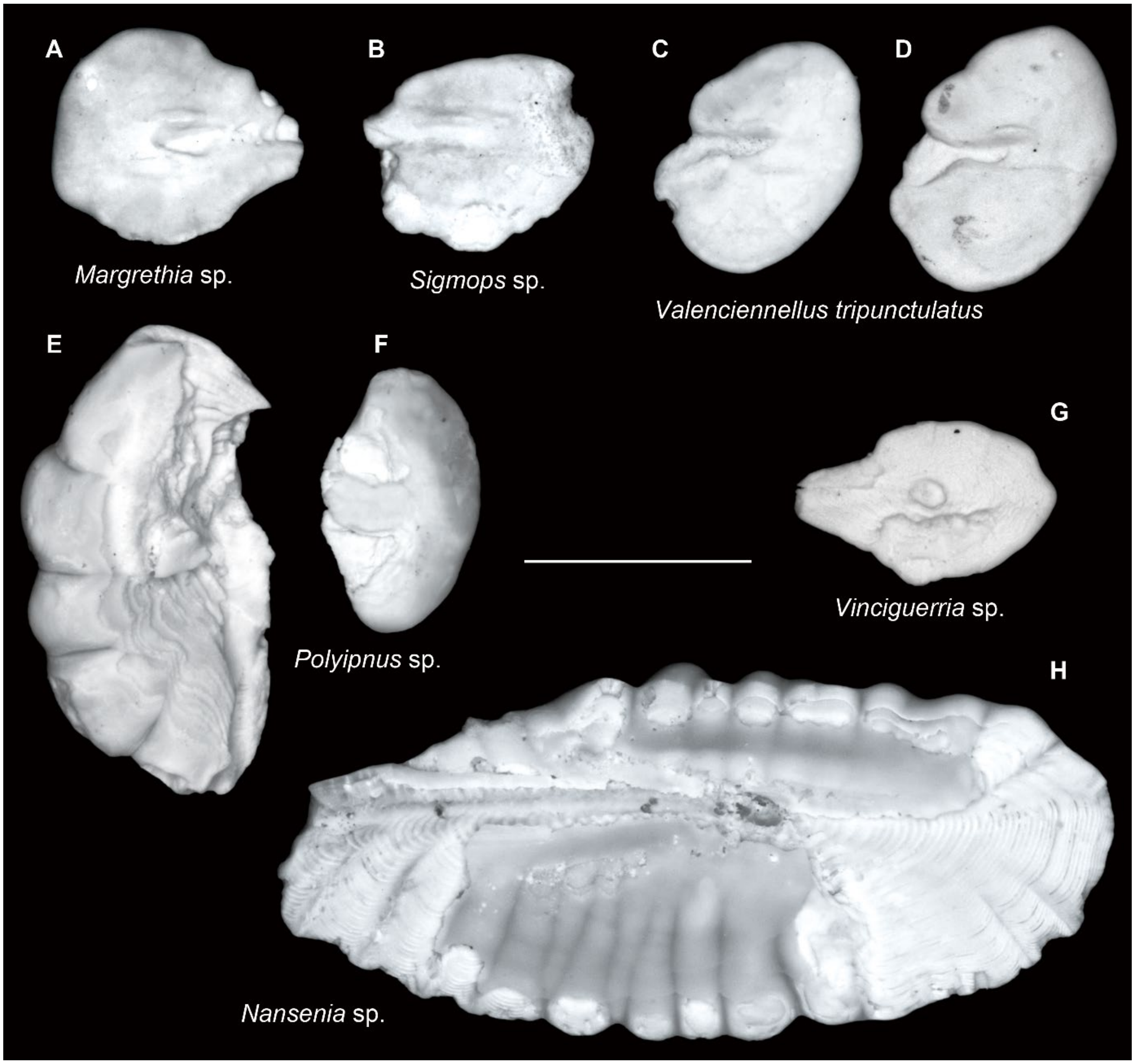

Fig. S12. Otoliths from ODP Hole 1115B. A, *Margrethia* sp., 1H3W, 120-122 cm; **B**, *Sigmops* sp., 1H1W, 65-67 cm; **C**, **D**, *Valenciennellus tripunctulatus* (Esmark, 1871), 1H1W, 50-52 cm; **E**, **F**, *Polyipnus* sp.; **E**, 1H4W, 30-32 cm; **F**, 1H5W, 55-57 cm; **G**, *Vinciguerria* sp., 2H1W, 120-122 cm; **H**, *Nansenia* sp., 1H2W, 90-92cm. Scale bar = 1 mm.

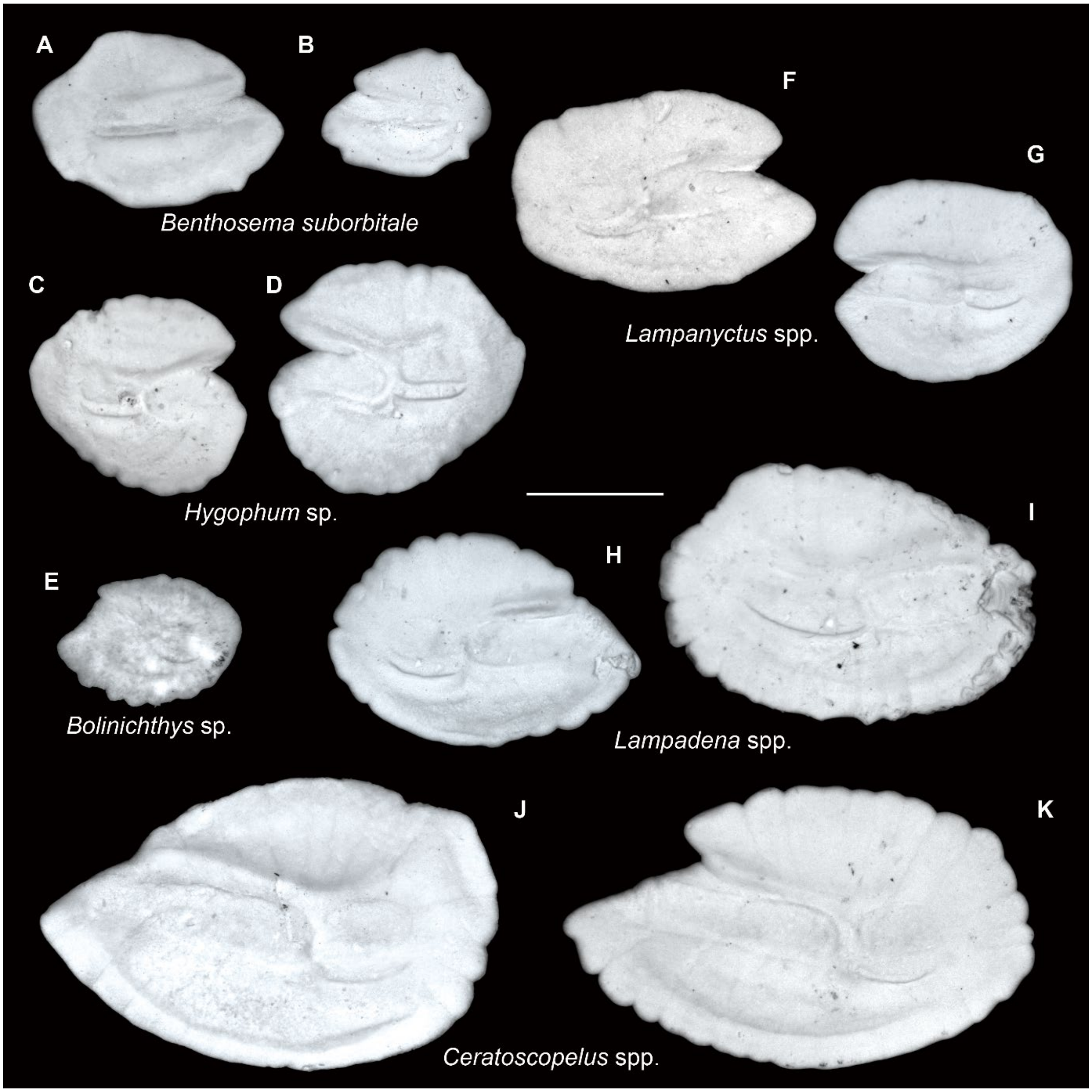

Fig. S13. Otoliths from ODP Hole 1115B. A, **B**, *Benthosema suborbitale* (Gilbert, 1913); **A**, 1H3W, 95-97 cm; **B**, 1H5W, 75-77 cm; **C**, **D**, *Hygophum* sp.; **C**, 2H4W, 110-112 cm; **D**, 1H4W, 45-47 cm; **E**, *Bolinichthys* sp., 1H1W, 30-32 cm; **F**, **G**, *Lampanyctus* spp.; **F**, 1H1W, 35-37 cm; **G**, 1H3W, 65-67 cm; **H**, **I**, *Lampadena* spp.; **H**, 1H2W, 90-92 cm; **I**, 1H2W, 55-57 cm; **J**, **K**, *Ceratoscopelus* spp.; **J**, 2H1W, 130-132 cm; **K**, 1H2W, 0-2 cm. Scale bar = 1 mm.

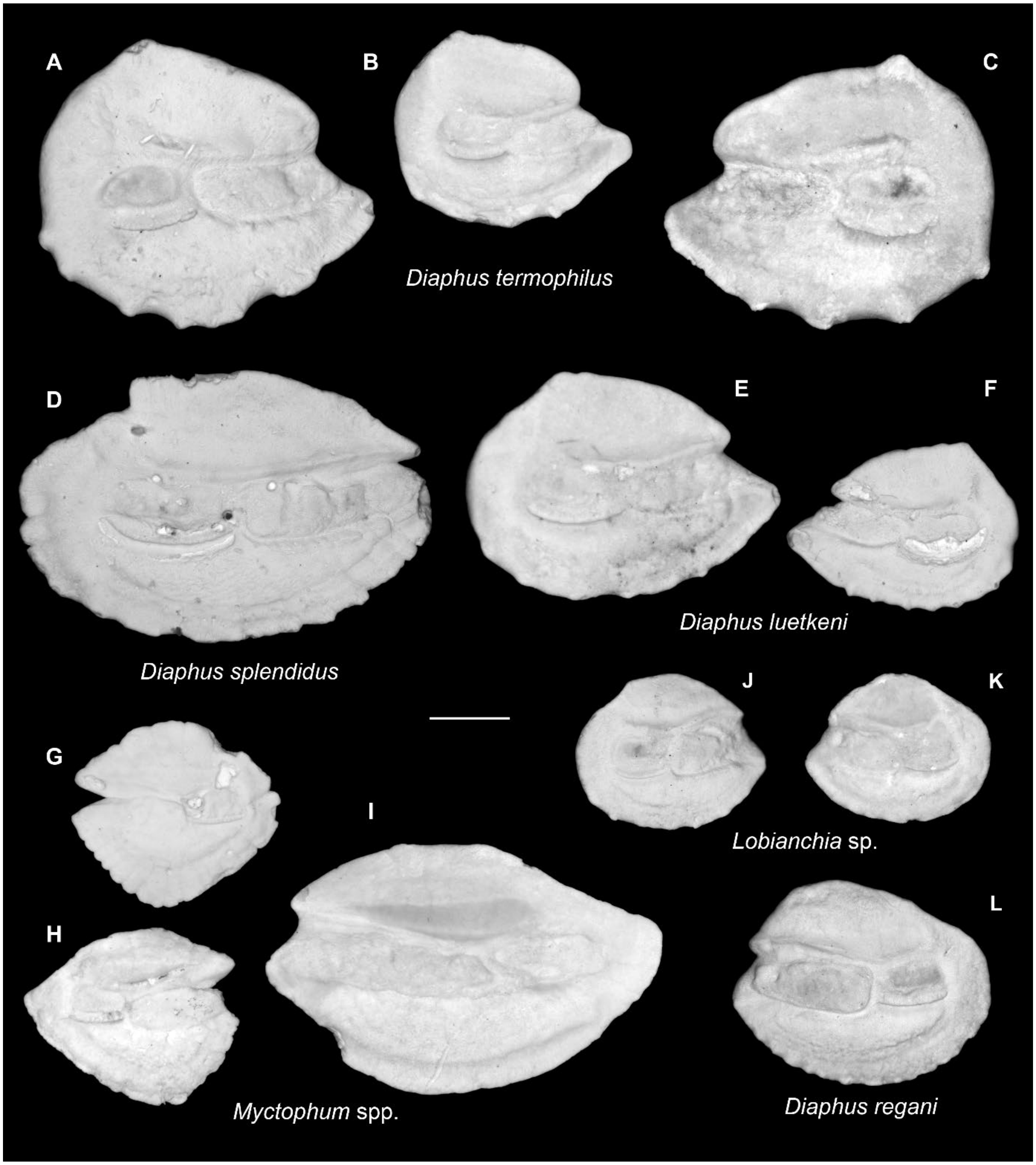

Fig. S14. Otoliths from ODP Hole 1115B. **A**-**C**, *Diaphus termophilus* Tåning, 1928; **A**, 1H2W, 90-92 cm; **B**, 1H4W, 25-27 cm; **C**, 1H4W, 110-112 cm; **D**, *Diaphus splendidus* (Brauer, 1904), 2H4W, 50-52 cm; **E**, **F**, *Diaphus luetkeni* (Brauer, 1904); **E**, 1H3W, 115-117 cm; **F**, 1H5W, 65-67 cm; **G**-**I**, *Myctophum* spp.; **G**, 1H2W, 125-127 cm; **H**, 1H3W, 145-147 cm; **I**, 1H1W, 75-77 cm; **J**, **K**, *Lobianchia* sp.; **J**, 1H3W, 85-87 cm; **K**, 1H2W, 125-127 cm; **L**, *Diaphus regani* Tåning, 1932, 1H3W, 70-72 cm. Scale bar = 1 mm.

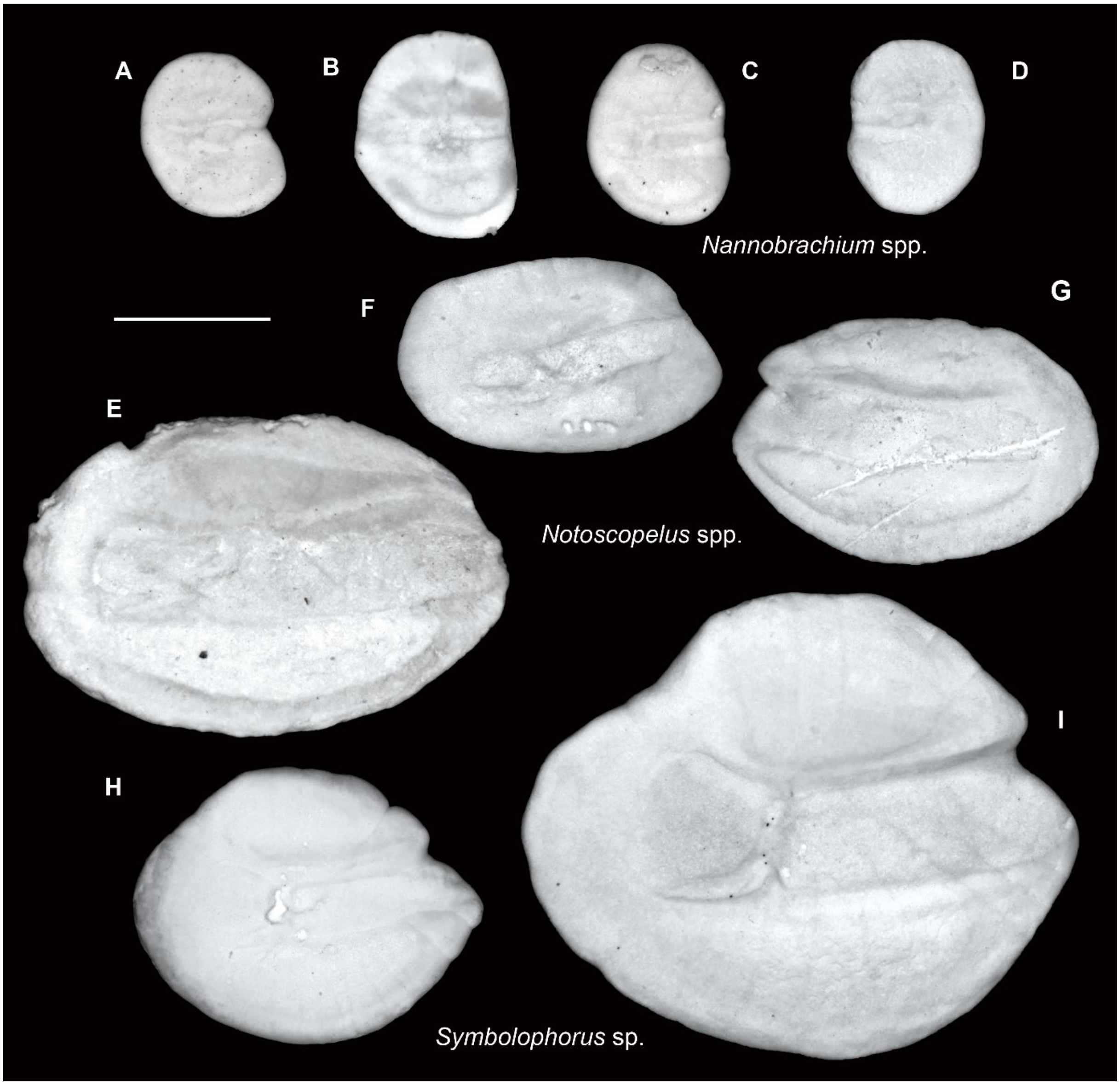

Fig. S15. Otoliths from ODP Hole 1115B. A-**D**, *Nannobrachium* spp.; **A**, 1H5W, 75-77 cm; **B**, 2H5W, 15-17 cm; **C**, 2H1W, 15-17 cm; **D**, 2H2W, 50-52 cm; **E**-**G**, *Notoscopelus* spp.; **E**, 1H4W, 140-142 cm; **F**, 2H2W, 130-132 cm; **G**, 2H6W, 85-87 cm; **H**, **I**, *Symbolophorus* sp.; **H**, 1H3W, 35-37 cm; **I**, 2H2W, 135-137 cm. Scale bar = 1 mm.

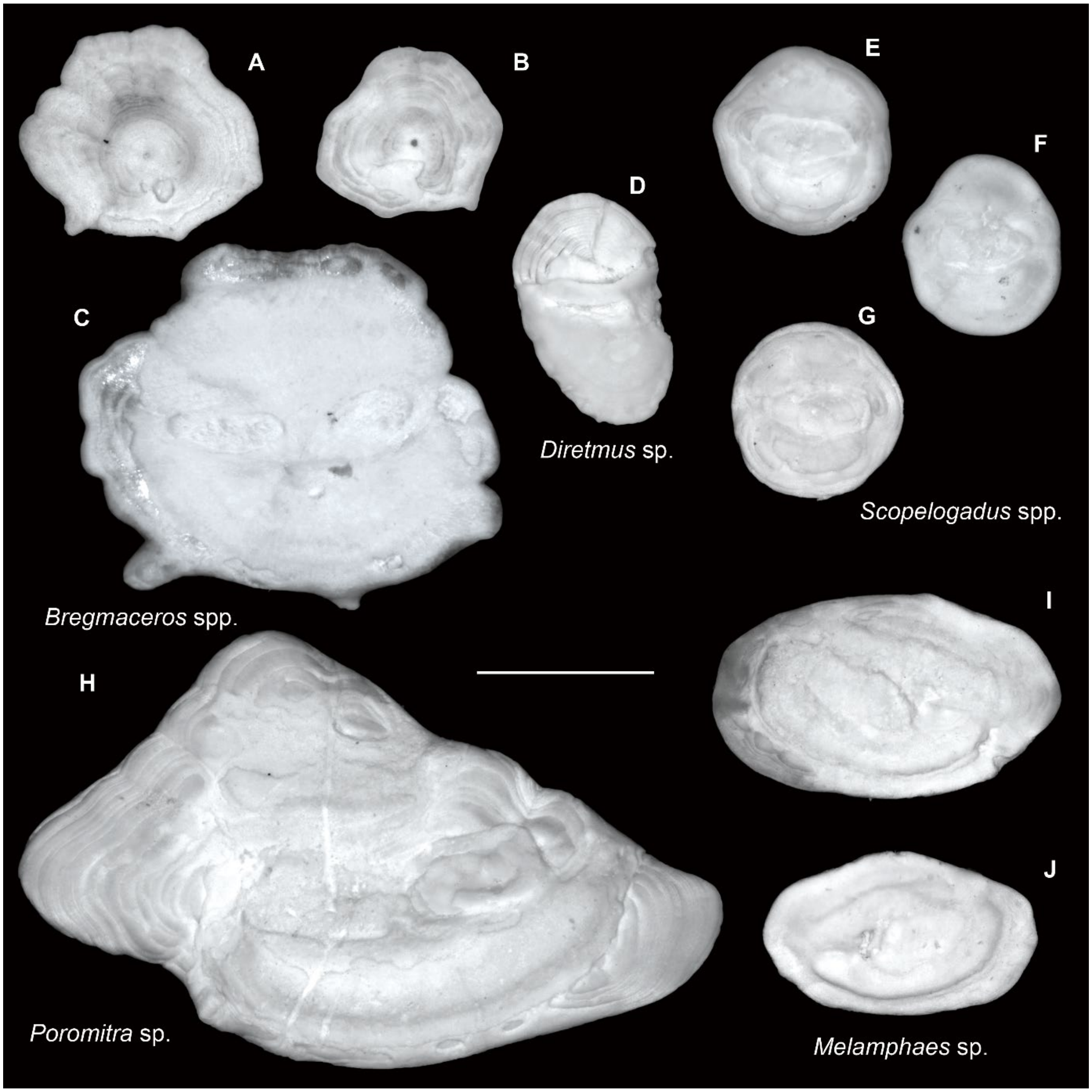

Fig. S16. Otoliths from ODP Hole 1115B. A-**C**, *Bregmaceros* spp.; **A**, 1H5W, 45-47 cm; **B**, 1H4W, 130-132 cm; **C**, 2H1W, 105-107 cm; **D**, *Diretmus* sp., 2H5W, 15-17 cm; **E**-**G**, *Scopelogadus* spp.; **E**, 1H4W, 130-132 cm; **F**, 1H3W, 60-62 cm; **G**, 1H3W, 70-72 cm; **H**, *Poromitra* sp., 2H2W, 145-147 cm; **I**, **J**, *Melamphaes* sp.; **I**, 1H3W, 120-122 cm; **J**, 1H2W, 140-142 cm. Scale bar = 1 mm.

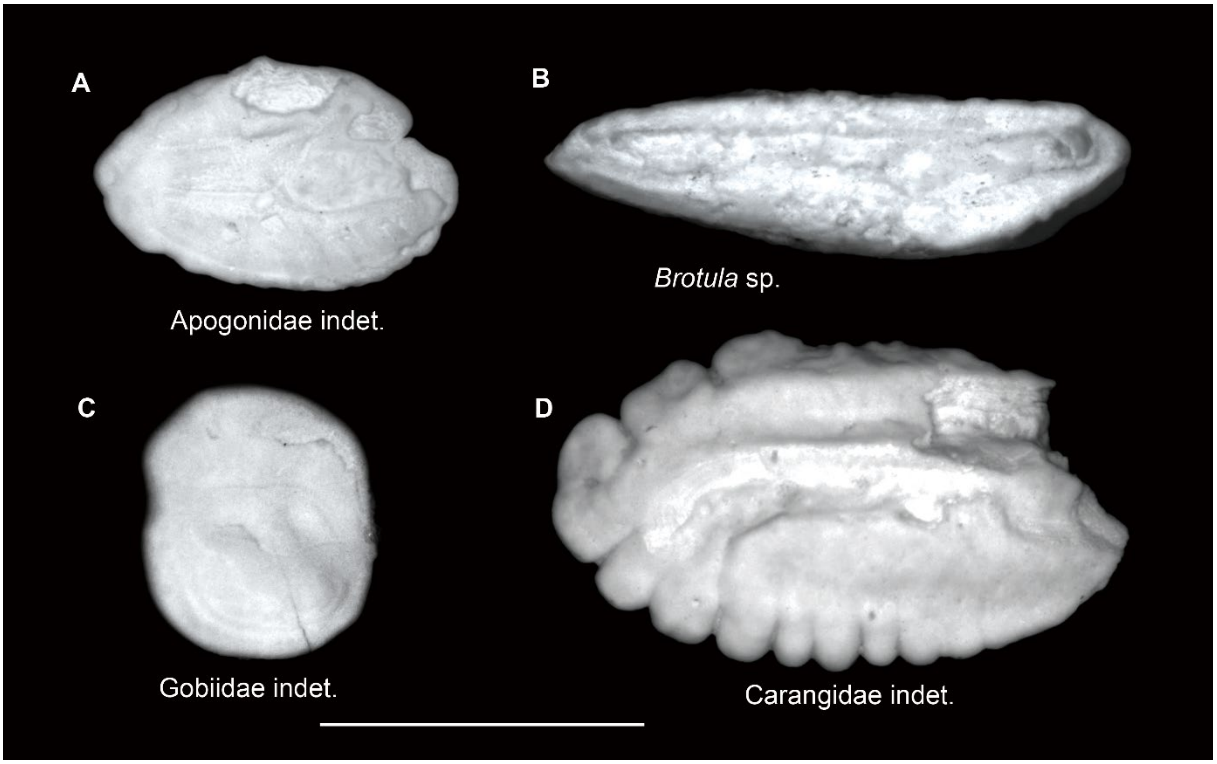

Fig. S17. Otoliths from ODP Hole 1115B. A, Apogonidae indet., 1H3W, 55-57 cm; **B**, *Brotula* sp., 1H1W, 45-47 cm; **C**, Gobiidae indet., 1H3W, 95-97 cm; **D**, Carangidae indet., 2H1W, 115-117 cm. Scale bar = 1 mm.

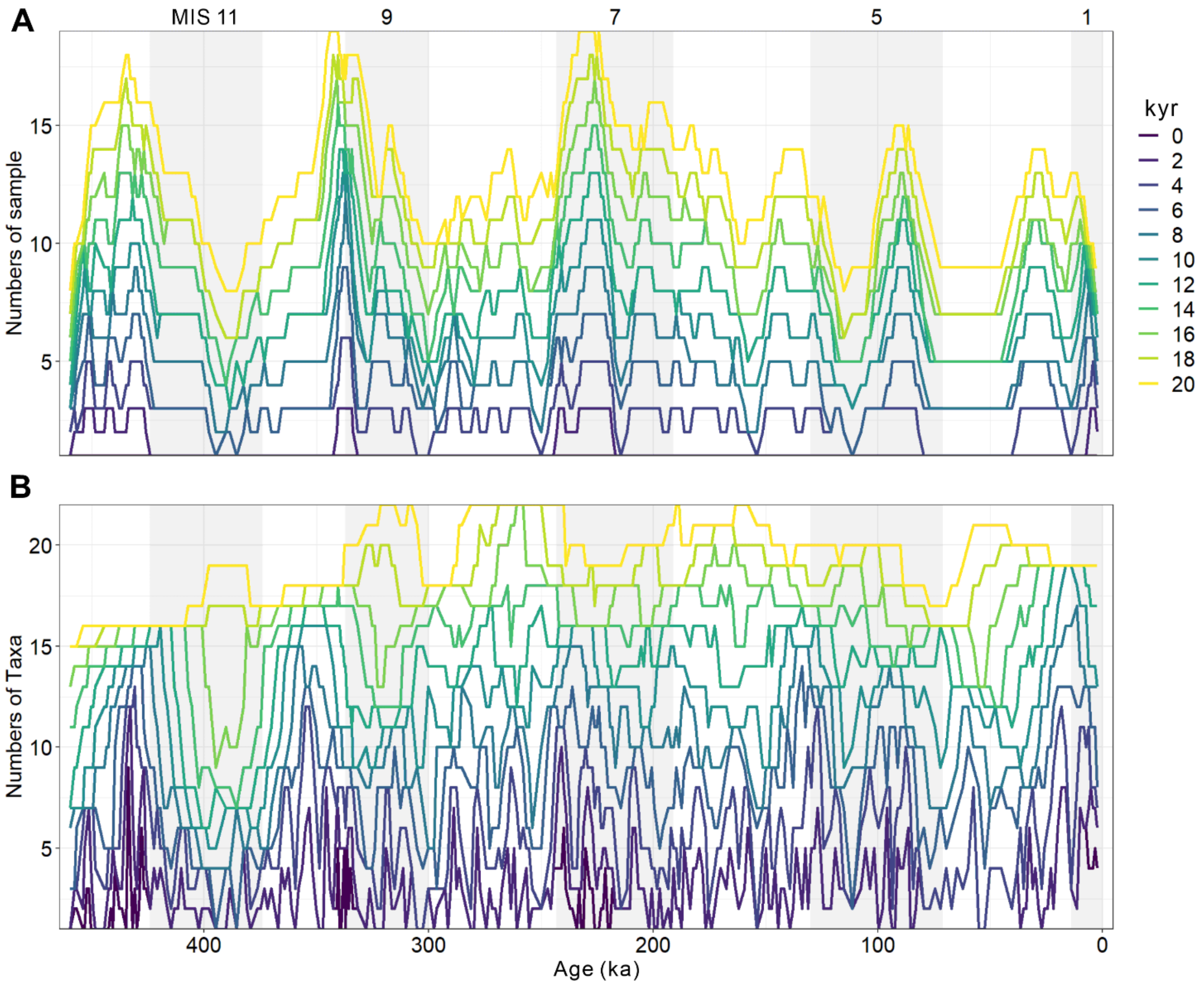

Fig. S18. Numbers of sample (A) and observed numbers of taxa (B) in each moving window. Gray rectangles indicate interglacial periods (Marine Isotope Stages 1, 5, 7, 9, 11 on the upper x-axis).

Data S1. (separate file)
Summary of each sample and results of otolith identification.

Data S2. (separate file)
Seawater temperature data (smoothed using a 10-ka moving window method) from core MD05-2925 (Materials and Methods).

Data S3. (separate file)
Weight of the otoliths and total coarse sands of each sample.

REFERENCES AND NOTES

1. S. L. C. Giering, R. Sanders, R. S. Lampitt, T. R. Anderson, C. Tamburini, M. Boutrif, M. V. Zubkov, C. M. Marsay, S. A. Henson, K. Saw, K. Cook, D. J. Mayor, Reconciliation of the carbon budget in the ocean's twilight zone. *Nature* **507**, 480–483 (2014).

2. X. Irigoien, T. A. Klevjer, A. Røstad, U. Martinez, G. Boyra, J. L. Acuña, A. Bode, F. Echevarria, J. I. Gonzalez-Gordillo, S. Hernandez-Leon, S. Agusti, D. L. Aksnes, C. M. Duarte, S. Kaartvedt, Large mesopelagic fishes biomass and trophic efficiency in the open ocean. *Nat. Commun.* **5**, 3271 (2014).

3. S. Hernández-León, R. Koppelmann, E. Fraile-Nuez, A. Bode, C. Mompeán, X. Irigoien, M. P. Olivar, F. Echevarría, M. L. Fernández de Puelles, J. I. González-Gordillo, A. Cózar, J. L. Acuña, S. Agustí, C. M. Duarte, Large deep-sea zooplankton biomass mirrors primary production in the global ocean. *Nat. Commun.* **11**, 6048 (2020).

4. G. K. Saba, A. B. Burd, J. P. Dunne, S. Hernández-León, A. H. Martin, K. A. Rose, J. Salisbury, D. K. Steinberg, C. N. Trueman, R. W. Wilson, S. E. Wilson, Toward a better understanding of fish-based contribution to ocean carbon flux. *Limnol. Oceanogr.* **66**, 1639–1664 (2021).

5. B. H. Robison, Conservation of deep pelagic biodiversity. *Conserv. Biol.* **23**, 847–858 (2009).

6. A. Martin, P. Boyd, K. Buesseler, I. Cetinic, H. Claustre, S. Giering, S. Henson, X. Irigoien, I. Kriest, L. Memery, C. Robinson, G. Saba, R. Sanders, D. Siegel, M. Villa-Alfageme, L. Guidi, The oceans' twilight zone must be studied now, before it is too late. *Nature* **580**, 26–28 (2020).

7. M. Hidalgo, H. I. Browman, Developing the knowledge base needed to sustainably manage mesopelagic resources. *ICES J. Mar. Sci.* **76**, 609–615 (2019).

8. R. Proud, M. J. Cox, A. S. Brierley, Biogeography of the global ocean's mesopelagic zone. *Curr. Biol.* **27**, 113–119 (2017).

9. D. Nolf, *Otolithi piscium* (Fischer, 1985).

10. D. Nolf, *The Diversity of Fish Otoliths, Past and Present* (Royal Belgian Institute of Natural Sciences, 2013).

11. W. Schwarzhans, G. Carnevale, The rise to dominance of lanternfishes (Teleostei: Myctophidae) in the oceanic ecosystems: A paleontological perspective. *Paleobiology* **47**, 446–463 (2021).

12. W. A. Jones, D. M. Checkley Jr., Mesopelagic fishes dominate otolith record of past two millennia in the Santa Barbara Basin. *Nat. Commun.* **10**, 4564 (2019).

13. C.-H. Lin, A. Girone, D. Nolf, Fish otolith assemblages from Recent NE Atlantic sea bottoms: A comparative study of palaeoecology. *Palaeogeogr. Palaeoclimatol. Palaeoecol.* **446**, 98–107 (2016).

14. C.-H. Lin, M. Taviani, L. Angeletti, A. Girone, D. Nolf, Fish otoliths in superficial sediments of the Mediterranean Sea. *Palaeogeogr. Palaeoclimatol. Palaeoecol.* **471**, 134–143 (2017).

15. C.-H. Lin, Y.-P. Chiang, V. M. Tuset, A. Lombarte, A. Girone, Late quaternary to recent diversity of fish otoliths from the red sea, central mediterranean, and NE Atlantic sea bottoms. *Geobios* **51**, 335–358 (2018).

16. E. C. Sibert, M. E. Zill, E. T. Frigyik, R. D. Norris, No state change in pelagic fish production and biodiversity during the Eocene–Oligocene transition. *Nat. Geosci.* **13**, 238–242 (2020).

17. R. Salvatteci, R. R. Schneider, E. Galbraith, D. Field, T. Blanz, T. Bauersachs, X. Crosta, P. Martinez, V. Echevin, F. Scholz, A. Bertrand, Smaller fish species in a warm and oxygen-poor Humboldt Current system. *Science* **375**, 101–104 (2022).

18. P. De Deckker, The Indo-Pacific Warm Pool: Critical to world oceanography and world climate. *Geosci. Lett.* **3**, 1–12 (2016).

19. Shipboard Scientific Party, Site 1115, in *Proceedings of the Ocean Drilling Program, Initial Reports* (1999), vol. 180; www-odp.tamu.edu/publications/180_IR/VOLUME/CHAPTERS/CHAP_09.PDF.

20. X.-H. Yan, C.-R. Ho, Q. Zheng, V. Klemas, Temperature and size variabilities of the Western Pacific Warm Pool. *Science* **258**, 1643–1645 (1992).

21. M. O. Hill, Diversity and evenness: A unifying notation and its consequences. *Ecology* **54**, 427–432 (1973).

22. A. Chao, Y. Kubota, D. Zelený, C.-H. Chiu, C.-F. Li, B. Kusumoto, M. Yasuhara, S. Thorn, C.-L. Wei, M. J. Costello, R. K. Colwell, Quantifying sample completeness and comparing diversities among assemblages. *Ecol. Res.* **35**, 292–314 (2020).

23. A. Chao, C.-H. Chiu, L. Jost, Unifying species diversity, phylogenetic diversity, functional diversity, and related similarity and differentiation measures through Hill numbers. *Annu. Rev. Ecol. Evol. Syst.* **45**, 297–324 (2014).

24. K. A. Jakob, P. A. Wilson, A. Bahr, C. T. Bolton, J. Pross, J. Fiebig, O. Friedrich, Plio-Pleistocene glacial-interglacial productivity changes in the eastern equatorial Pacific upwelling system. *Paleoceanography* **31**, 453–470 (2016).

25. L. Lo, S.-P. Chang, K.-Y. Wei, S.-Y. Lee, T.-H. Ou, Y.-C. Chen, C.-K. Chuang, H.-S. Mii, G. S. Burr, M.-T. Chen, Y.-H. Tung, M.-C. Tsai, D. A. Hodell, C.-C. Shen, Nonlinear climatic sensitivity to greenhouse gases over past 4 glacial/interglacial cycles. *Sci. Rep.* **7**, 4626 (2017).

26. L. Lo, C.-C. Shen, C. Zeeden, Y.-H. Tsai, Q. Yin, C.-C. Yang, T.-L. Chang, Y.-C. Su, H.-S. Mii, C.-K. Chuang, Y.-C. Chem, Orbital control on the thermocline structure during the past 568 kyr in the Solomon Sea, southwest equatorial Pacific. *Quat. Sci. Rev.* **295**, 107756 (2022)

27. L. E. Lisiecki, M. E. Raymo, A Pliocene-Pleistocene stack of 57 globally distributed benthic $\delta^{18}O$ records. *Paleoceanography* **20**, PA1003 (2005).

28. R. A. Feely, C. L. Sabine, K. Lee, F. J. Millero, M. F. Lamb, D. Greeley, J. L. Bullister, R. M. Key, T.-H. Peng, A. Kozyr, T. Ono, C. S. Wong, In situ calcium carbonate dissolution in the Pacific Ocean. *Global Biogeochem. Cycles* **16**, 91-1–91-12 (2002).

29. C.-K. Chuang, L. Lo, C. Zeeden, Y.-M. Chou, K.-Y. Wei, C.-C. Shen, H.-S. Mii, Y.-P. Chang, Y.-H. Tung, Integrated stratigraphy of ODP Site 1115 (Solomon Sea, southwestern equatorial Pacific) over the past 3.2 Ma. *Mar. Micropaleontol.* **144**, 25–37 (2018).

30. K. Agiadi, M. Azzarone, Q. Hua, D. S. Kaufman, D. Thivaiou, P. G. Albano, The taphonomic clock in fish otoliths. *Paleobiology* **48**, 154–170 (2022).

31. A. Paytan, M. Kastner, F. P. Chavez, Glacial to interglacial fluctuations in productivity in the Equatorial Pacific as indicated by marine Barite. *Science* **274**, 1355–1357 (1996).

32. J. H. Connell, Diversity in tropical rain forests and coral reefs. *Science* **199**, 1302–1310 (1978).

33. K. Rehfeld, T. Münch, S. L. Ho, T. Laepple, Global patterns of declining temperature variability from the Last Glacial Maximum to the Holocene. *Nature* **554**, 356–359 (2018).

34. D. G. Boyce, D. P. Tittensor, B. Worm, Effects of temperature on global patterns of tuna and billfish richness. *Mar. Ecol. Prog. Ser.* **355**, 267–276 (2008).

35. M. Yasuhara, C.-L. Wei, M. Kucera, M. J. Costello, D. P. Tittensor, W. Kiessling, T. C. Bonebrake, C. R. Tabor, R. Feng, A. Baselga, K. Kretschmer, B. Kusumoto, Y. Kubota, Past and future decline of tropical pelagic biodiversity. *Proc. Natl. Acad. Sci. U.S.A.* **117**, 12891–12896 (2020).

36. T. H. Boag, W. Gearty, R. G. Stockey, Metabolic tradeoffs control biodiversity gradients through geological time. *Curr. Biol.* **31**, 2906–2913.e3 (2021).

37. C. Chaudhary, A. J. Richardson, D. S. Schoeman, M. J. Costello, Global warming is causing a more pronounced dip in marine species richness around the equator. *Proc. Natl. Acad. Sci. U.S.A.* **118**, e2015094118 (2021).

38. J. B. C. Jackson, Ecological extinction and evolution in the brave new ocean. *Proc. Natl. Acad. Sci. U.S.A.* **105**, 11458–11465 (2008).

39. N. R. Geraldi, G. T. Kellison, N. M. Bacheler, Climate indices, water temperature, and fishing predict broad scale variation in fishes on temperate reefs. *Front. Mar. Sci.* **6**, 30 (2019).

40. G. L. Britten, E. C. Sibert, Enhanced fish production during a period of extreme global warmth. *Nat. Commun.* **11**, 5636 (2020).

41. R. Vaquer-Sunyer, C. M. Duarte, Temperature effects on oxygen thresholds for hypoxia in marine benthic organisms. *Glob. Chang. Biol.* **17**, 1788–1797 (2011).

42. R. Salvatteci, D. Field, D. Gutiérrez, T. Baumgartner, V. Ferreira, L. Ortlieb, A. Sifeddine, D. Grados, A. Bertrand, Multifarious anchovy and sardine regimes in the humboldt current system during the last 150 years. *Glob. Chang. Biol.* **24**, 1055–1068 (2018).

43. R. F. Anderson, M. Q. Fleisher, Y. Lao, G. Winckler, Modern $CaCO_3$ preservation in equatorial Pacific sediments in the context of late-Pleistocene glacial cycles. *Mar. Chem.* **111**, 30–46 (2008).

44. L. Lo, C.-C. Shen, K.-Y. Wei, G. S. Burr, H.-S. Mii, M.-T. Chen, S.-Y. Lee, M.-C. Tsai, Millennial meridional dynamics of the Indo-Pacific Warm Pool during the last termination. *Clim. Past* **10**, 2253–2261 (2014).

45. T. Qu, S. Gao, R. A. Fine, Subduction of South Pacific Tropical Water and its equatorward pathways as shown by a simulated passive tracer. *J. Phys. Oceanogr.* **43**, 1551–1565 (2013).

46. P. Anand, H. Elderfield, M. H. Conte, Calibration of Mg/Ca thermometry in planktonic foraminifera from a sediment trap time series. *Paleoceanogr. Paleoclimatol.* **18**, 1050 (2003).

47. C.-H. Lin, C.-W. Chang, *Otolith Atlas of Taiwan Fishes* (National Museum of Marine Biology and Aquarium, 2012).

48. M. J. Smale, G. Watson, T. Hecht, Otolith atlas of southern African marine fishes. *Ichtyol. Monogr.* **1**, 1–253 (1995).

49. J. Rivaton, P. Bourret, *Les otolithes des poissons de l'Indo-Pacifique* (Institut de recherche pour le développement, 1999).

50. W. Schwarzhans, A comparative morphological study of the Recent otoliths of the genera *Diaphus*, *Idiolychnus* and *Lobianchia* (Myctophidae). *Palaeo Ichthyologica* **13**, 41–82 (2013).

51. W. Schwarzhans, F. Ohe, Lanternfish otoliths (Teleostei, Myctophidae) from the Pliocene and Pleistocene of Japan. *Riv. Ital. Paleontol. Stratigr.* **125**, 355–400 (2019).

52. J. C. Herguera, W. H. Berger, Paleoproductivity from benthic foraminifera abundance: Glacial to postglacial change in the west-equatorial Pacific. *Geology* **19**, 1173 (1991).

53. L. Diester-Haass, P. A. Meyers, L. Vidal, The late Miocene onset of high productivity in the Benguela Current upwelling system as part of a global pattern. *Mar. Geol.* **180**, 87–103 (2002).

54. K. A. Jakob, S. L. Ho, A. N. Meckler, J. Pross, J. Fiebig, F. Keppler, O. Friedrich, Stable biological production in the eastern Equatorial Pacific across the Plio-Pleistocene transition (~3.35–2.0 Ma). *Paleoceanogr. Paleoclimatol.* **36**, e2020PA003965 (2021).

55. P. Howell, N. Pisias, J. Balance, J. Baughman, L. Ochs, *ARAND time-series analysis software* (Brown University, 2006).

56. J. Laskar, P. Robutel, F. Joutel, M. Gastineau, A. C. M. Correia, B. Levrard, A long-term numerical solution for the insolation quantities of the Earth. *Astron. Astrophys. Suppl. Ser.* **428**, 261–285 (2004).

57. A. Chao, L. Jost, *Diversity Analysis* (Chapman & Hall/CRC, 2010).

58. A. M. Ellison, Partitioning diversity. *Ecology* **91**, 1962–1963 (2010).

59. P. Legendre, E. D. Gallagher, Ecologically meaningful transformations for ordination of species data. *Oecologia* **129**, 271–280 (2001).

60. M. Schulz, M. Mudelsee, REDFIT: Estimating red-noise spectra directly from unevenly spaced paleoclimatic time series. *Comput. Geosci.* **28**, 421–426 (2002).

61. R. A. Locarnini, A. V. Mishonov, J. I. Antonov, T. P. Boyer, H. E. Garcia, Temperatures, in *World Ocean Atlas*, S. Levitus, Ed. (U.S. Government Printing, 2006), vol. 1, pp. 182.
